# Cyclin-Dependent Kinase CRK9, Required for Spliced Leader *trans* Splicing of Pre-mRNA in Trypanosomes, Functions in a Complex with a New L-Type Cyclin and a Kinetoplastid-Specific Protein

**DOI:** 10.1371/journal.ppat.1005498

**Published:** 2016-03-08

**Authors:** Nitika Badjatia, Sung Hee Park, Daniela L. Ambrósio, Justin K. Kirkham, Arthur Günzl

**Affiliations:** Department of Genetics and Genome Sciences, University of Connecticut Health Center, Farmington, Connecticut, United States of America; University of California, Los Angeles, UNITED STATES

## Abstract

In eukaryotes, cyclin-dependent kinases (CDKs) control the cell cycle and critical steps in gene expression. The lethal parasite *Trypanosoma brucei*, member of the phylogenetic order Kinetoplastida, possesses eleven CDKs which, due to high sequence divergence, were generically termed CDC2-related kinases (CRKs). While several CRKs have been implied in the cell cycle, CRK9 was the first trypanosome CDK shown to control the unusual mode of gene expression found in kinetoplastids. In these organisms, protein-coding genes are arranged in tandem arrays which are transcribed polycistronically. Individual mRNAs are processed from precursor RNA by spliced leader (SL) *trans* splicing and polyadenylation. CRK9 ablation was lethal in cultured trypanosomes, causing a block of *trans* splicing before the first transesterification step. Additionally, *CRK9* silencing led to dephosphorylation of RNA polymerase II and to hypomethylation of the SL cap structure. Here, we tandem affinity-purified CRK9 and, among potential CRK9 substrates and modifying enzymes, discovered an unusual tripartite complex comprising CRK9, a new L-type cyclin (CYC12) and a protein, termed CRK9-associated protein (CRK9AP), that is only conserved among kinetoplastids. Silencing of either *CYC12* or *CRK9AP* reproduced the effects of depleting CRK9, identifying these proteins as functional partners of CRK9 *in vivo*. While mammalian cyclin L binds to CDK11, the CRK9 complex deviates substantially from that of CDK11, requiring CRK9AP for efficient CRK9 complex formation and autophosphorylation *in vitro*. Interference with this unusual CDK rescued mice from lethal trypanosome infections, validating CRK9 as a potential chemotherapeutic target.

## Author Summary

Kinetoplastids are vector-borne, unicellular parasites that cause devastating human diseases in the tropics and subtropics of the world. Interestingly, kinetoplastids express protein-coding genes differently than other eukaryotes by producing polycistronic pre-mRNAs that require processing into individual mRNAs by spliced leader *trans* splicing and polyadenylation. While each parasite mRNA is *trans-*spliced, this particular splicing process is absent in mammalian and insect hosts of kinetoplastids. We recently discovered that the cyclin-dependent kinase (CDK) CRK9 of the kinetoplastid *Trypanosoma brucei* is essential for *trans* splicing and parasite viability. Due to the essential roles of CDKs in cell proliferation, CDK inhibition is an established strategy against cancer, suggesting that chemotherapeutic intervention of CRK9 will halt proliferation of kinetoplastid parasites in their hosts. To enable inhibitor studies of CRK9, we have characterized the CRK9 enzyme complex and discovered a new cyclin and a kinetoplastid-specific protein, both of which are essential for the formation of active CRK9. The tripartite nature of the CRK9 complex and sequence insertions that disrupt both kinase and cyclin domains suggest that CRK9 deviates structurally from human CDKs. Finally, by demonstrating that CRK9 ablation prevented trypanosomes from establishing lethal infections in mice, we validated CRK9 as a potential anti-parasitic drug target.

## Introduction

*Trypanosoma brucei*, *Trypanosoma cruzi* and *Leishmania* spp. are unicellular, vector borne, human parasites belonging to the early-diverged phylogenetic order Kinetoplastida whose hallmark, the kinetoplast, is a network of catenated mitochondrial DNA. Kinetoplastid parasites collectively affect millions of people worldwide primarily in developing countries, causing both debilitating and fatal human diseases. There is no preventive vaccine for these diseases and the current medications are unsatisfactory due to poor efficacy, toxicity and developing drug resistance [1]. Trypanosomatid parasites share a unique mode of protein coding gene expression that is distinct from their hosts. Their genomes are organized in large gene clusters of tandemly linked protein coding genes which are transcribed polycistronically. Individual mRNAs are resolved from pre-mRNA by spliced leader (SL) *trans* splicing and polyadenylation. In SL *trans* splicing, the SL, derived from the small nuclear SL RNA, is spliced onto the 5^/^ end of each mRNA [2, 3]. Like *cis* splicing, e.g. the removal of introns, SL *trans* splicing comprises two transesterifications, generating a Y-shaped structural intermediate after the first splicing step that is analogous to the lariat structure in *cis* splicing [4, 5]. The 39 nt-long SL harbors an extensively modified cap structure, called cap4, that consists of a 7-methylguanosine cap nucleotide followed by four methylated nucleotides [6]. Cap4 is important for SL *trans* splicing [7, 8] and for efficient translation [9]. Since each and every mRNA carries a SL, *trans* splicing is absolutely essential for kinetoplastid viability. In *T*. *brucei*, the cdc2-related kinase 9 (CRK9) appears to be of crucial importance for this unusual mode of gene expression since depletion of this cyclin-dependent kinase (CDK) led to a lethal block of *trans* splicing in both the insect-stage procyclic (PF) and the mammalian-infective bloodstream form (BF) of the parasite [10].

CDKs are serine/threonine kinases that require association with a cyclin for enzymatic activity; they are characterized by an ATP binding pocket, a cyclin-binding PSTAIRE-like helix domain and an activating T-loop. They were first identified as key regulators of cell cycle progression and were subsequently found to have important roles in gene transcription and RNA processing [11]. Although individual CDKs can function in both realms, they have been divided into cell cycle-related and “transcriptional” CDKs [12]. Furthermore, CDKs of both groups were found to have additional roles in the cell, for example in regulating DNA repair and proteolysis [13]. While CDKs that are important for the cell cycle are regulated by their sequential binding to different cyclins, each of which exhibit distinct cell cycle-dependent expression patterns, transcriptional CDKs and their cyclins are expressed throughout the cell cycle, typically forming a single CDK-cyclin complex. In mammals, CDK7 has a dual role in both cell cycle and transcription. Uniquely, CDK7 forms a trimeric complex with cyclin H and the RING finger protein MAT1 (ménage a trois 1) that phosphorylates and activates cell cycle CDKs in their T-loops and, therefore, has been termed the CDK-activating kinase (CAK). CAK is also part of the basal transcription factor TFIIH and, in this association, phosphorylates the carboxy-terminal domain of RPB1 (CTD), the largest subunit of RNA polymerase (pol) II, during transcription initiation, facilitating promoter clearance of the enzyme and recruitment of RNA processing factors to the CTD including the capping enzyme [14]. In murine and human cells, CDK11, which mainly occurs as two isoforms, CDK11^p110^ and CDK11^p58^, is another CDK with a dual role. While CDK11^p58^ is expressed from a G2/M-specific internal ribosome entry site of the *CDK11* mRNA and is implicated in mitosis [15–18], CDK11^p110^ is constitutively expressed and involved in transcription and pre-mRNA splicing [19]. CDK11 is of ancient origin since orthologues have been found in protozoa; it is present in all metazoans and the fission yeast *Schizosaccharomyces pombe* but absent from budding yeast *Saccharomyces cerevisiae* [20, 21]. Across systems, CDK11 forms an enzyme complex with L-type cyclins whose hallmark is a C-terminal RS domain. Consistent with the importance of this domain in spliceosomal serine/arginine-rich (SR) and SR-like proteins and the functional role of CDK11, L-type cyclins were found to be important in RNA splicing [22–24].

Due to their crucial importance for gene expression and cell proliferation, CDKs are considered to be promising drug targets, most notably for anti-cancer therapy to inhibit cell proliferation. There are currently 11 CDK inhibitors under clinical evaluation and, recently, the first such drug was approved for the treatment of metastatic breast cancer [25, 26]. *Trypanosoma brucei* harbors eleven CDKs, as many as in flies and 3–5 more than in fungi [12], indicating a high dependence of this unicellular organism on CDK-mediated control. Trypanosome CDKs, however, are divergent in sequence from their eukaryotic counterparts, preventing their unequivocal classification. Thus, trypanosome CDKs were generically termed CRK1-4 and CRK6-12. In addition, ten cyclins (CYC2-11) have been identified in *T*. *brucei* so far [27, 28]. RNA interference (RNAi)-mediated gene silencing experiments identified CRK1-3, CRK6, CRK9 and CRK12 as essential for trypanosome viability [10, 29–34]. Less is known about the complex composition and specific functions of these enzymes. CRK3 was shown to be the functional homolog of human CDK1/yeast CDC2; it partners with CYC6 (also known as cyclin B2) and, as does its counterpart, regulates mitosis controlling G2/M progression [33, 35, 36]. Moreover, CRK3 of the related organism *Leishmania major* was able to complement a *S*. *pombe* cdc2 null mutant [37]. Interestingly, this CDK also interacts with P12CKS1, a non-cyclin homolog of the human CDK regulatory subunit CKS1 [38]. *T*. *brucei* CRK1 was shown to be important for G1/S progression and interacted with the four PH80 cyclins CYC2, CYC4, CYC5 and CYC7 *in vitro* and *in vivo* [33, 39, 40]. CRK2, CRK4 and CRK6 appear to have accessory functions in the cell cycle although their cyclin partners are unknown [41]. Recently, CYC9 was co-isolated with CRK12 in tandem affinity purification. Both CYC9 and CRK12 were essential in BFs, but depletion of either protein resulted in distinct phenotypes, suggesting that while CYC9 is important for cytokinesis, CRK12 has a role in endocytosis [42].

CRK9 is the first trypanosome CDK known to have a dual role in the cell cycle and in gene expression. In a pioneering study, ablation of CRK9 in PF trypanosomes affected mitosis and led to defects in kinetoplast and basal body segregation during cytokinesis [29]. CRK9’s essential function in gene expression became apparent by RNA analysis of *CRK9*-silenced PFs and BFs, both of which revealed a strong decline of mature mRNA levels and a concomitant increase of unspliced pre-mRNA. Moreover, while the *trans* splicing substrate SL RNA was dramatically increased in *CRK9*-silenced cells, the Y structure splicing intermediate exhibited a strong decline in abundance. Since introduction of an RNAi-resistant *CRK9* wild-type gene into these cells completely rescued this phenotype, whereas introduction of an equivalent *CRK9* gene carrying a mutation in its T-loop sequence did not, these results unequivocally demonstrated that CRK9 enzyme activity is crucial for the first step of SL *trans* splicing in trypanosomes [10]. Further defects observed upon *CRK9* silencing were the loss of RPB1 phosphorylation, and a hypomethylation of the SL cap. Surprisingly, the effects on mRNA abundance, RPB1 phosphorylation and cap4 formation seen upon *CRK9* silencing were quantitatively reproduced by depleting a subunit of the spliceosomal PRP19 complex that, in other systems, was shown to be essential for spliceosome activation, strongly indicating a direct role of CRK9 in RNA splicing [43].

As a prerequisite for inhibition studies of this crucially important CDK, we have characterized the CRK9 enzyme complex and validated CRK9 enzyme activity as a potential drug target in the mouse model. We found that CRK9 forms a tripartite complex together with a novel, previously unannotated L-type cyclin that we named CYC12 and a kinetoplastid-specific protein that we termed CRK9-associated protein or CRK9AP. *CYC12* and *CRK9AP* silencing reproduced the *CRK9* silencing phenotypes, identifying them as essential, functional partners of CRK9 *in vivo*. Moreover, formation of a recombinant enzyme complex that was capable of autophosphorylation required the co-expression of all three proteins in wheat germ extract, confirming the tripartite nature of the enzyme complex. This is highly unusual as, with the exception of CDK7, CDKs function as CDK-cyclin heterodimers.

## Results

### Two un-annotated proteins co-purify and co-precipitate with CRK9

**Fig 1:**
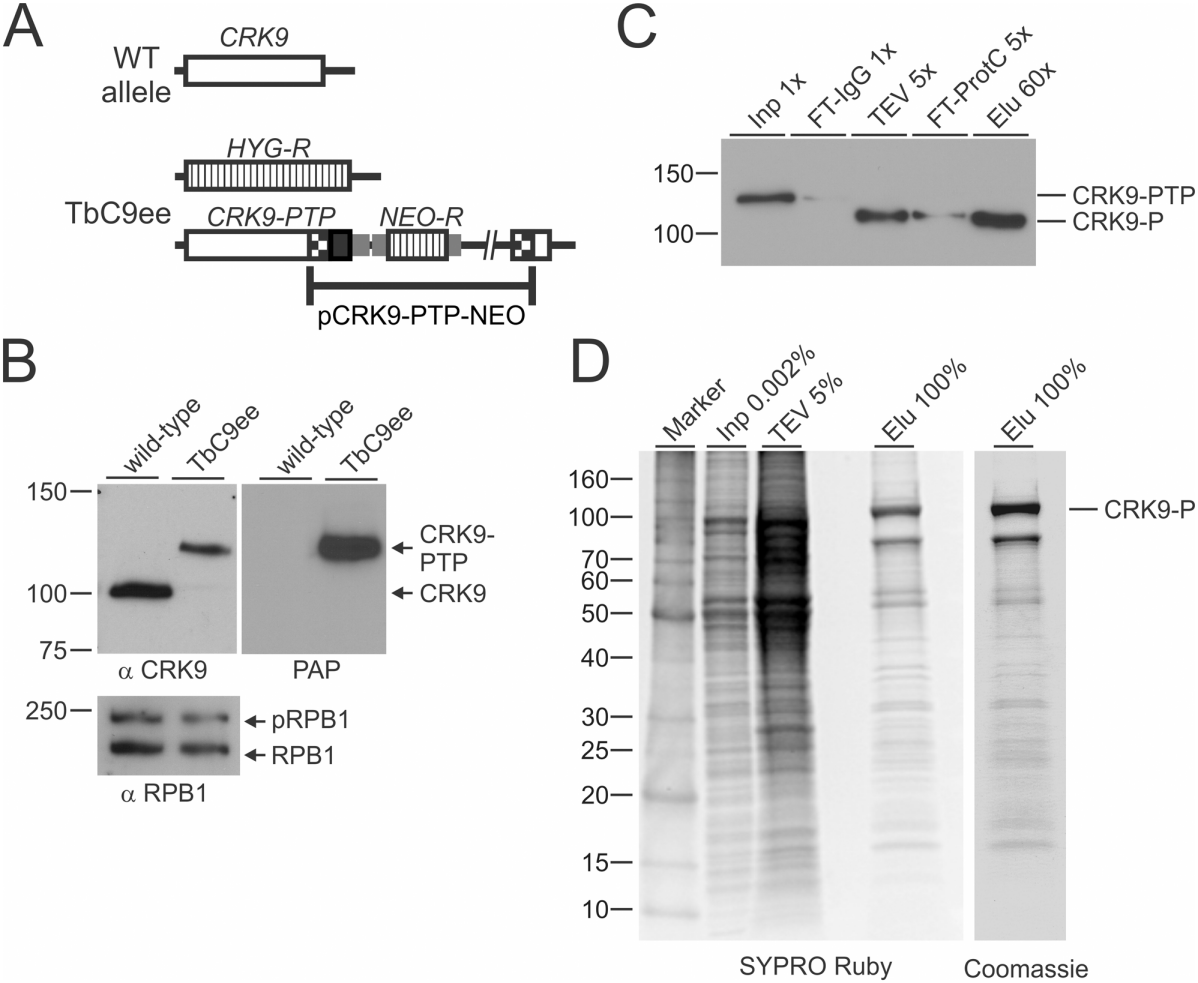
Tandem affinity purification of CRK9. (A) Schematic depiction (not to scale) of the CRK9 locus in procyclic TbC9ee cells that exclusively express CRK9-PTP and no untagged CRK9. In these cells one wild-type CRK9 allele (open box) was knocked out by a hygromycin phosphotransferase gene (*HYG-R*, striped box). Integration of plasmid CRK9-PTP-NEO introduced the PTP sequence (black box) and the neomycin phosphotransferase (*NEO-R*) to the second allele. Smaller gray boxes indicate gene flanks for RNA processing signals and checkered boxes depict CRK9 sequences encoded in the plasmid. (B) Immunoblot of whole cell lysates of wild-type trypanosomes and of TbC9ee cells, detecting CRK9 and CRK9-PTP, respectively, with the newly generated anti-CRK9 polyclonal immune serum. On the same blot, phosphorylated (p) and unphosphorylated RPB1 was detected as a loading control with a polyclonal antibody, and CRK9-PTP specifically with the peroxidase anti-peroxidase reagent (PAP) that binds to the ProtA domains of the PTP tag. (C) Immunoblot monitoring of the CRK9-PTP purification detecting CRK9-PTP in crude extract (Inp) and the flowthrough of IgG affinity chromatography (FT-IgG), and CRK9-P in TEV protease eluate, the flowthrough of the anti-ProtC immunoaffinity chromatography (FT-ProtC) and the final eluate (Elu) with the monoclonal HCPC4 anti-ProtC antibody. The x-values indicate relative amounts analyzed. (D) Protein analysis of the CRK9-PTP purification. Proteins of crude extract, the TEV eluate and the final eluate were separated on 10–20% SDS polyacrylamide gel and first stained with SYPRO Ruby and, subsequently, with Coomassie blue (right panel). Marker sizes in kDa are indicated on the left.

In order to tandem affinity-purify CRK9 from trypanosome extract, we generated the clonal PF cell line TbC9ee which expresses C-terminally PTP-tagged CRK9 and no untagged CRK9. The PTP tag is a composite tag consisting of the protein C epitope (ProtC), a tobacco etch virus (TEV) protease cleavage site and tandem protein A domains (ProtA) [44]. The cell line was obtained by two consecutive transfections, in which one *CRK9* allele was replaced by the hygromycin phosphotransferase gene (*HYG-R*) and one allele modified by targeted insertion of plasmid CRK9-PTP-NEO, which fused the PTP coding sequence to the 3^/^ end of the CRK9 coding region (Fig 1A). As a tool to study CRK9 expression, we raised a specific immune serum in rats against a recombinant, GST-tagged CRK9 protein fragment that was expressed and purified from *Escherichia coli*. The immune serum detected a single protein band of ~100 kDa in wild-type PF lysates which is slightly larger than the predicted CRK9 mass of 85.6 kDa, whereas in TB9Cee lysates this band was replaced by a ~120 kDa band consistent with the ~19 kDa mass of the PTP tag (Fig 1B). Since the latter band was specifically detected by a ProtA-specific probe, these results confirmed exclusive expression of CRK9-PTP in TbC9ee cells. Since TbC9ee cells did not exhibit reduced culture growth as compared to wild-type cells and the phosphorylation status of RPB1, which has been linked to CRK9 function, was comparable in wild-type and TbC9ee cells (Fig 1B, bottom panel), we concluded that the PTP tag did not interfere with CRK9 function.

Facilitated by the PTP tag, we purified CRK9-PTP consecutively by IgG affinity chromatography, TEV protease cleavage and anti-ProtC immunoaffinity chromatography. An anti-ProtC immunoblot analysis revealed efficient capture of the tagged protein in all purification steps (Fig 1C). Separation of the final eluate and, as controls, of the input material and the TEV protease fraction, by SDS-PAGE and staining of the proteins by SYRPRO Ruby and Coomassie Blue, revealed a dominant protein band of ~105 kDa, representing CRK9-P (note that CRK9-PTP was reduced by ~15 kDa to CRK9-P by TEV protease cleavage), and, unexpectedly, many co-purified protein bands (Fig 1D). Liquid chromatography-tandem mass spectrometry (LC/MS/MS) of two independent CRK9-PTP purifications revealed a total of 162 proteins that were identified with an expect value smaller than 0.01 and a minimum of two unique peptides (S1 Table). The majority of proteins, including those with very high protein scores, were components of both ribosomal subunits. This massive co-purification of ribosomal proteins was unexpected since CRK9 is a nuclear protein that is not enriched in the nucleolus [29], and since previous PTP purifications of various nuclear complexes contained only a few, low scoring ribosomal proteins. Since the functional association of CRK9 with ribosomal complexes remains unclear, we focused on non-ribosomal proteins. Table 1 lists the most significant identifications. While no annotated cyclin was detected in the purification, the protein with the second highest score is a new trypanosome cyclin (see below) which we termed CYC12 (accession number Tb927.10.9160). Among the top scoring proteins are three dual specificity protein kinases capable of phosphorylating aliphatic serines and threonines as well as aromatic tyrosines, indicating that they are involved in CRK9-dependent regulatory pathways. Two of them (Tb927.7.3880 and Tb927.10.350) represent dual-specificity tyrosine phosphorylation-regulated kinases (DYRKs) which, in other systems, were shown to regulate the cell cycle by altering protein turnover rates [45] whereas the third enzyme appears to be a CDC-like kinase (CLK; domain PKc_CLK, accession cd14134, E = 5.9e^-57^) which is distinct from the recently characterized kinetochore CLKs KKT10 and KKT19 [46] and which, as with several CLKs in other systems, may directly function in RNA splicing [47]. Furthermore, co-purification of the spliceosomal SR protein TSR1 [48], which is phosphorylated at multiple sites [49], may represent a direct link between CRK9 and the splicing machinery. Finally, the list of CRK9-associated proteins contains three that appear to be conserved only within the genus *Trypanosoma*, suggesting that, in addition to CRK9’s fundamental role in gene expression, there may be additional, trypanosome-specific functions associated with this kinase.

**Table 1:** Mass spectrometric identification of CRK9 co-purified proteins.

Rank	Annotation^1^	Accession #^1^	*M*_r_ (kDa)	Score^2^	% Coverage	emPAI^3^
**1**	**CRK9**	**Tb927.2.4510**	**85.5**	**13,418**	**73.9**	**10.3**
**2**	**CYC12**	**Tb927.10.9160**	**71.2**	**9,956**	**43.3**	**8.1**
6	DYRK protein kinase ^4^	Tb927.7.3880	87.0	1,797	49.7	2.1
19	hypothetical, conserved ^6^	Tb927.9.13970	36.8	805	47.3	2.8
22	DYRK protein kinase ^4^	Tb927.10.350	58.6	795	35.5	1.5
24	PABP2	Tb927.9.10770	62.2	724	37.8	1.7
28	putative CLK kinase ^5^	Tb927.3.1610	74.2	661	34.1	1.0
**37**	**CRK9AP**	**Tb927.3.4170**	**13.0**	**568**	**37.2**	**5.5**
46	hypothetical, conserved	Tb927.3.3740	69.1	525	34.4	0.8
50	hypothetical, conserved ^6^	Tb927.1.4680	37.3	474	36.3	1.2
59	hypothetical, conserved	Tb927.10.11600	66.0	382	21.7	0.7
62	MRB1-assoc. protein	Tb927.11.6320	53.2	353	29.3	0.6
69	NRBD1 (RNA-binding)	Tb927.11.14000	28.8	299	37.8	1.7
73	hypothetical, conserved ^6^	Tb927.7.3080	47.3	285	31.9	1.0
74	RACK1 (kinase receptor)	Tb927.11.11360	34.7	285	18.7	1.0
77	hypothetical, conserved	Tb927.4.3150	38.1	275	27.7	0.7
84	PNO1 (pre-rRNA process.)	Tb927.9.11840	24.1	257	32.5	1.3
93	TSR1, splicing factor	Tb927.8.900	37.4	226	22.1	0.6

List of CRK9-PTP co-purified proteins, identified by LC/MS/MS, that were not a common contaminant of previous tandem affinity purifications, not annotated a ribosomal protein, had a Mascot Score of at least 200 and an emPAI value equal or greater than 0.5. The rank number is according to the complete protein list in Table A in S1 Text. The CRK9 complex subunits are specified by bold lettering.

^**1**^ Protein annotation and accession numbers are from the *T*. *b*. *brucei* 927 database at www.TriTrypDB.org, although proteins were identified through the *T*. *b*. *brucei* 427 Lister genome database whose annotation and assembly is not as complete.

^**2**^ The Mascot Score corresponds to -10 x LOG_10_(P), where P is the absolute probability that the observed match is a random event.

^**3**^ The emPAI (**e**xponentially **m**odified **P**rotein **A**bundance **I**ndex) is a measure for the relative amount of an identified protein in the final eluate [86].

^**4**^ DYRK: Dual-specificity tyrosine phosphorylation-regulated kinase.

^**5**^ CLK: CDC-like kinase; dual specificity kinase.

^**6**^ These proteins appear to be conserved only in the genus *Trypanosoma*.

**Fig 2:**
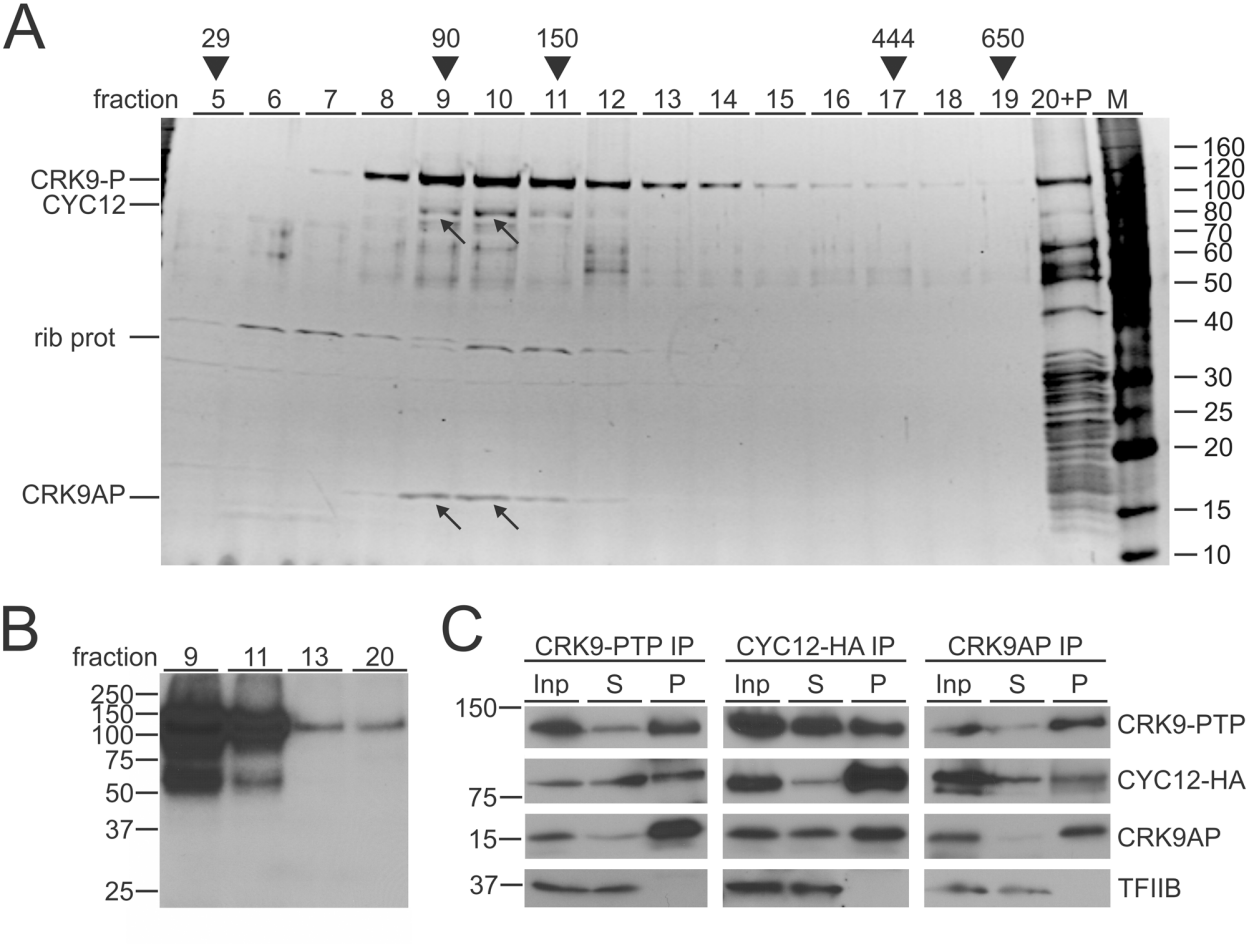
CRK9 interacts and co-sediments with two unannotated proteins. (A) CRK9-PTP tandem affinity-purified material was sedimented through a 10–40% linear sucrose gradient by ultracentrifugation and fractionated into 20 aliquots from top to bottom. Note that pelleted proteins were resuspended in fraction 20 (20+P). Proteins from each fraction were separated by SDS-PAGE and stained with SYPRO Ruby. Protein bands were excised and identified by LC/MS/MS. Arrows point to the CYC12 and CRK9AP bands which co-sediment with CRK9 in fractions 9/10. The 35 kDa band with a peak in fractions 10/11 was found to be the putative ribosomal protein L5 (Tb927.9.15110/15150). (B) Kinase assay with materials from indicated fractions suggest autophosphorylation of CRK9. (C) Reciprocal co-IP assays of extracts prepared from a cell line in which CRK9 was exclusively PTP-tagged and an HA tag sequence was inserted at the 3’end of one CYC12 allele. The precipitate (P) was loaded at a fourfold excess relative to extract (Inp) and supernatant (S). Detection of the RNA pol II transcription factor TFIIB served as a negative precipitation control.

To separate the actual kinase complex from co-purifying proteins, we sedimented the eluate of a CRK9-PTP purification through a linear sucrose gradient by ultracentrifugation. While part of the enzyme and nearly all other co-purified proteins migrated to the bottom of the gradient, CRK9-P exhibited a strong sedimentation peak in fractions 9–11 (Fig 2A). Protein bands of ~80, 37 and 15 kDa co-sedimented in these fractions. Since transcriptional CDKs of other systems are capable of autophosphorylating their T-loop motifs, we carried out a kinase assay with fractions 9, 11, 13, and 20, detecting disproportionately high CRK9-P phosphorylation in fractions 9 and 11, suggesting that fractions 9–11 contain an active CRK9 complex (Fig 2B). Mass spectrometry identified the protein of the 37 kDa band, which exhibited a slightly different sedimentation profile than the other two bands, as a putative ribosomal protein (Tb927.11.2050); it may be a direct interactor of CRK9 within the ribosome. The two other bands were non-ribosomal proteins: The 80 kDa and 15 kDa bands revealed CYC12 and CRK9AP (Tb927.3.4170), respectively, the latter being conserved only among kinetoplastid organisms (S1 Fig). To verify these protein identifications by reciprocal co-immunoprecipitation (co-IP), we C-terminally tagged CYC12 with HA in TbC9ee cells and raised a polyclonal immune serum in rats against recombinant, GST-tagged CRK9AP (S2 Fig). As shown in Fig 2C, precipitation of either CRK9-PTP, CYC12-HA or CRK9AP specifically co-precipitated the other two proteins, strongly indicating the presence of a tripartite kinase complex.

### CYC12 is a new L-type cyclin

**Fig 3:**
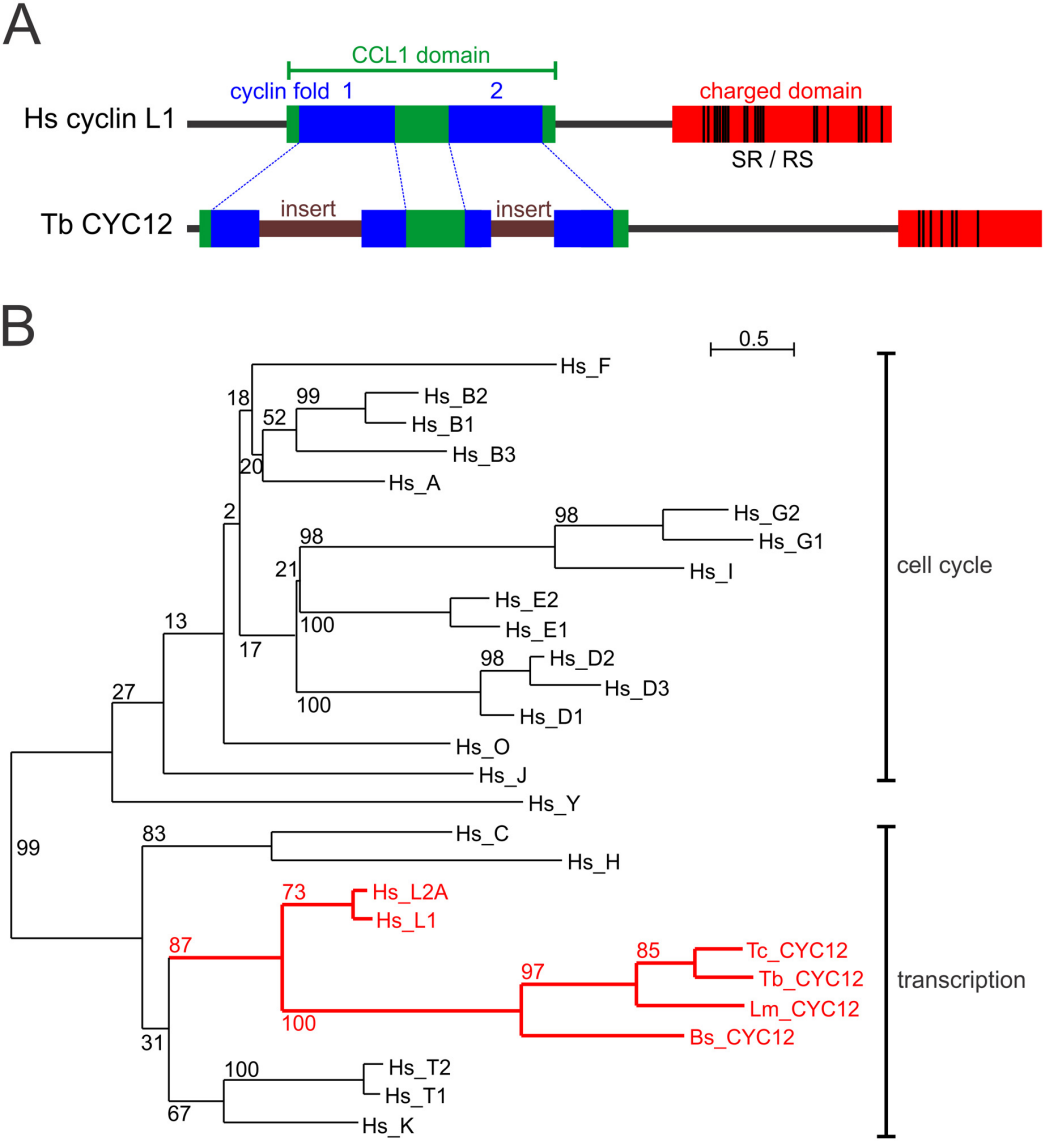
Cyclin CYC12 is an L-type cyclin. (A) Schematic drawing to scale of the human L1 and *T*. *brucei* CYC12 cyclins. The two cyclin folds (blue) are embedded in the CCL1 domain (green). The charged RS domain (red) was defined by a hydrophilicity Kyte & Doolittle blot score of < -2. Black lines indicate SR or RS dipeptides. Both cyclin domains of CYC12 are disrupted by insertions. (B) Phylogenetic tree, generated by the maximum likelihood algorithm and based on a multiple sequence alignment of the cyclin domains of human cyclins and of CYC12s from *T*. *brucei* (Tb), *T*. *cruzi* (Tc), *L*. *major* (Lm) and the bodonid *Bodo saltans* (Bs) (for accession numbers see S5 Fig). Cyclins involved in the cell cycle and in transcriptional control are indicated. Bootstrap values are indicated in percentages and were derived from 1000 replicates. The common branch of human cyclins L and kinetoplastid CYC12s is drawn in red.

Mammalian cyclin L homologues exclusively partner with CDK11. There are two human cyclin L paralogs, L1 and L2, which share 60% identity. These proteins have a ~200 amino acid-long N-terminal composite CCL1 domain that comprises the highly conserved Cyclin_N domain, the less conserved Cyclin_C domain and additional sequence conservation, characteristic for transcriptional cyclins, around these domains. In addition, cyclin L is an SR-related protein that features a conserved, C-terminal, highly positively charged arginine/serine-rich RS domain with repetitive “SR” dipeptide motifs (Fig 3A). A standard protein-protein BLAST search of the human proteome with the *T*. *brucei* CYC12 sequence returned L1 and L2 cyclins (E = 2e^-09^) but no other cyclins. A multiple sequence alignment of the CCL1 domain of cyclin L orthologs from model organisms and of kinetoplastid CYC12 sequences showed convincing sequence conservation across the whole domain, although, as expected, the Cyclin_C domain was less well conserved (S3 Fig). Interestingly, both cyclin domains were disrupted by substantial kinetoplastid-specific sequence insertions (in *T*. *brucei*, 74 aa and 45 aa long) which likely prevented recognition of CYC12 as a cyclin in trypanosomatid genome annotations and of the cyclin domains by the NCBI BLAST algorithm (Fig 3A, S3 Fig). Analysis of the C-terminus of CYC12 revealed a moderate accumulation of SR dipeptide motifs when compared to the human cyclin L domain (21 versus 7). However, conserved among all kinetoplastid CYC12 orthologs, the C-terminal domain has a similarly high isoelectric point of ~12, comparable to its counterparts in other eukaryotes.

To substantiate the notion that CYC12 is an L-type cyclin, we generated a phylogenetic tree with the alignment of all human cyclin sequences and four representative kinetoplastid CYC12 sequences. To avoid a potential bias of the charged cyclin L/CYC12 C-terminal domain, we restricted the analysis to the CCL1 domain. As expected, CYC12 sequences unambiguously partitioned with transcriptional cyclins and formed a branch with human L cyclins with a bootstrap value of 87% (Fig 3B). A more extensive phylogenetic analysis of complete sequence alignments of cyclins from model organisms and known kinetoplastid CYC12 orthologs revealed a similar tree, though the bootstrap value of the cyclin L/CYC12 cluster was reduced to 53% (S4 Fig). Taken together, these data strongly indicate that kinetoplastid CYC12 represents an L-type cyclin.

### CYC12 and CRK9AP are functional partners of CRK9

**Fig 4:**
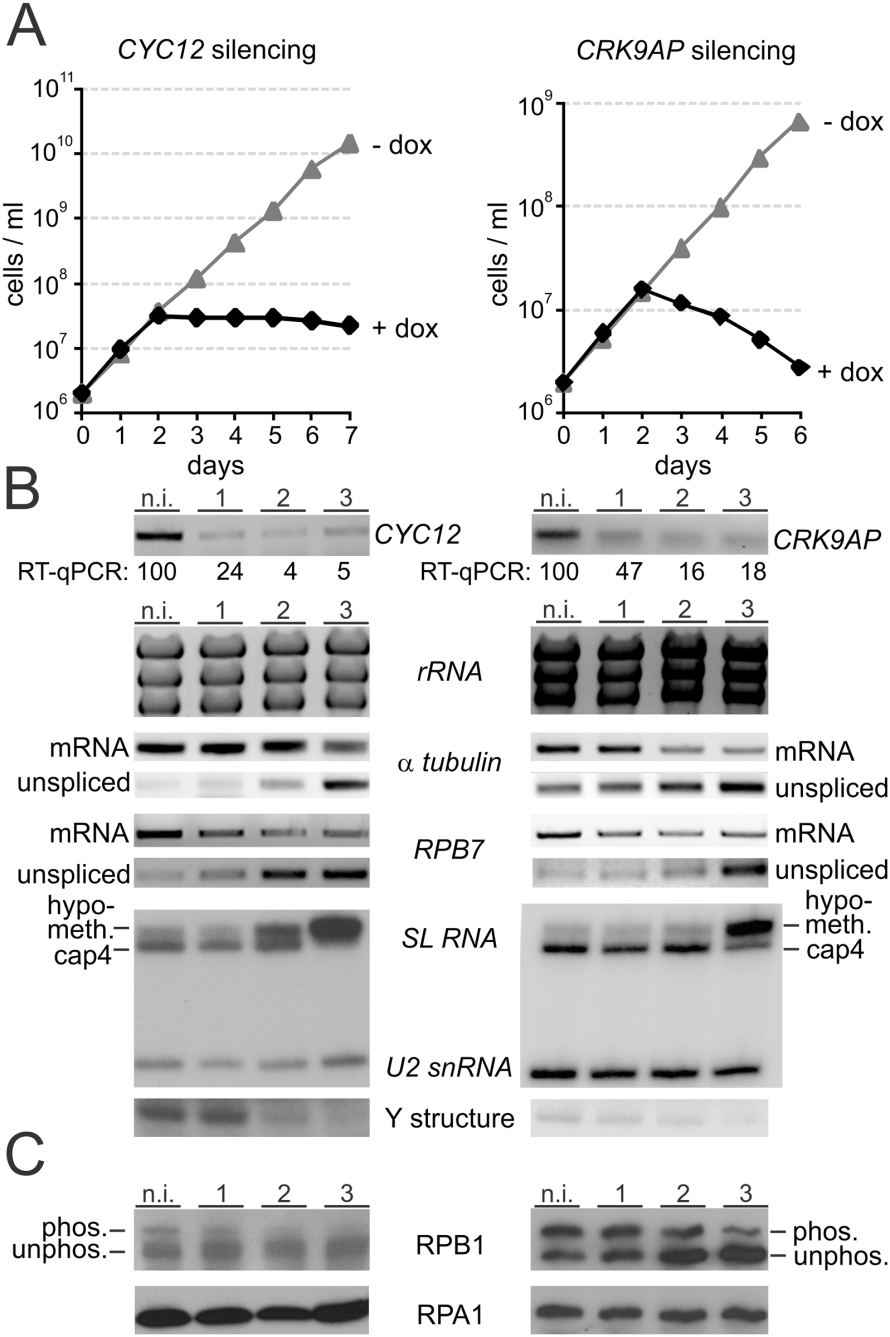
CYC12 and CRK9AP are functional partners of CRK9. (A) Cumulative culture growth curves were obtained for *CYC12* and *CRK9AP* silencing in the absence and presence of doxycycline (dox), the gene knockdown-inducing compound. For each knockdown a representative growth curve is shown. (B) Analysis of total RNA prepared from non-induced cells and cells in which *CYC12* or *CRK9AP* were silenced for 1, 2 or 3 days. *CYC12* or *CRK9AP* mRNA as well as α tubulin and *RPB7* mRNA were analyzed by reverse transcription of oligo-dT and semi-quantitative PCR, whereas unspliced, pre-mRNA of α tubulin and *RPB7* were analyzed by reverse transcription of random hexamers and by PCR using an oligonucleotide upstream of the SL addition site. rRNA was visualized by ethidium bromide staining after separation in an agarose gel. SL RNA, U2 snRNA and the Y structure intermediate were detected by primer extension assays using a SL RNA and a U2 snRNA-specific primer in the same reactions. (C) Anti-RPB1 immunoblot analysis of whole-cell lysates prepared from *CRK9AP*-silenced cells. Detection of the similar-sized RNA pol I subunit RPA1 served as a loading control.

To test whether CYC12 and CRK9AP are as important to trypanosome gene expression as CRK9, we first generated PF cell lines for conditional silencing of each gene. In these cells, addition of doxycycline induced the expression of *CYC12* or *CRK9AP* hairpin RNAs which, via the RNAi pathway, cause the degradation of their target mRNAs. While it was straightforward to obtain clonal cell lines for *CRK9AP* silencing, several rounds of transfections generated only a single line for the *CYC12* knockdown, suggesting that the *CYC12* expression level in PFs is critically important and vulnerable to a minor level of background expression of the dsRNA gene cassette in the absence of doxycyline. Nevertheless, the culture of this cell line stopped growing two days after induction, indicating a deleterious effect upon doxycycline induction (Fig 4A, left panel). We determined culture growth for two *CRK9AP* knockdown cell lines and observed a rapid halt of growth after two days of induction and a steady decline of cell numbers thereafter (Fig 4A, right panel and S5 Fig). RNA analysis of *CYC12*- and *CRK9AP*-silenced cells showed that both knockdowns were efficient, with *CYC12* and *CRK9AP* mRNA levels being reduced to 4% and 16%, respectively, after 2 days of induction (Fig 4B, top panels). In comparison, the onset of the growth defect in both *CYC12*- and *CRK9AP*-silenced cells was delayed by one day when compared to the *CRK9* knockdown [10]. In addition, while the number of *CRK9AP*-silenced cells rapidly declined, comparable to *CRK9*-silenced cells, CYC12 depletion caused cell numbers to remain constant, indicating a different dynamic of the lethal defect. When we generated corresponding knockdown cell lines in BF trypanosomes, this phenotypic variation was not observed and silencing of each of the three genes affected cell proliferation after 1 day of induction and eliminated live cells within 4 days of induction (S5 and S6 Figs) [10]. Thus, we concluded that *CYC12* and *CRK9AP* are essential genes in PF and BF *T*. *brucei*.

The *trans* splicing block upon *CRK9* silencing was revealed by a decrease of mature mRNA and the Y structure intermediate, the concomitant increase of unspliced pre-mRNA, and the accumulation of SL RNA with a hypomethylated cap structure [10]. Analyses of total RNA from PF *CYC12*- and *CRK9AP*-silenced cells showed corresponding defects (Fig 4B). As analyzed for α tubulin and *RPB7* (RPB7 encodes a subunit of RNA pol II) by semi-quantitative RT-PCR, mature mRNA declined upon gene silencing whereas the abundance of the corresponding unspliced pre-mRNA from these genes clearly increased during this period. Furthermore, primer extension assays of the same RNA preparations showed that, in both knockdowns, SL RNA increased, predominantly in its hypomethylated form, whereas the Y structure intermediate declined during the time course of the experiments. These results are consistent with a block of *trans* splicing before the first transesterification step. Analysis of α tubulin [pre-]mRNA levels in BFs confirmed the *trans* splicing defect in this life cycle stage (S6B Fig).

*CRK9* silencing also led to a loss of phosphorylation of the RNA pol II subunit RPB1 [10]. Correspondingly, immunoblotting of lysates from *CYC12*- and *CRK9A*P-silenced cells with anti-RPB1 immune serum revealed a decrease of the upper RPB1 band (Fig 4C), which previously was shown to contain phosphorylated RPB1 [10]. The loss of RPB1 phosphorylation was faster and more pronounced in BFs: after only day of induction, phosphorylated RPB1 was nearly undetectable in *CRK9*- and *CYC12*-silenced cells whereas it took two days for *CRK9AP*-silenced BFs to exhibit a reduction of this posttranslational modification (S6C Fig).

Finally, as described previously, all PFs that survive 3 days of *CRK9* silencing exhibit an atypical rounding up in culture [10, 29] reminiscent of FAT cells that were observed in the discovery of the RNA interference pathway upon tubulin gene knockdowns [50]. Silencing of *CYC12* and of *CRK9AP* resulted in the same characteristic phenotype (S7 Fig).

Taken together, *CYC12* and *CRK9AP* silencing resulted in the same block of *trans* splicing and RPB1 dephosphorylation in both PFs and BFs as observed previously upon *CRK9* silencing. Moreover, all three gene knockdowns caused widespread rounding up of PFs, an atypical death phenotype in this life cycle stage. Thus, we conclude that CRK9, CYC12 and CRK9AP are functional partners in facilitating SL *trans* splicing in trypanosomes.

### *CRK9AP* silencing caused rapid co-loss of CRK9 and CYC12

**Fig 5:**
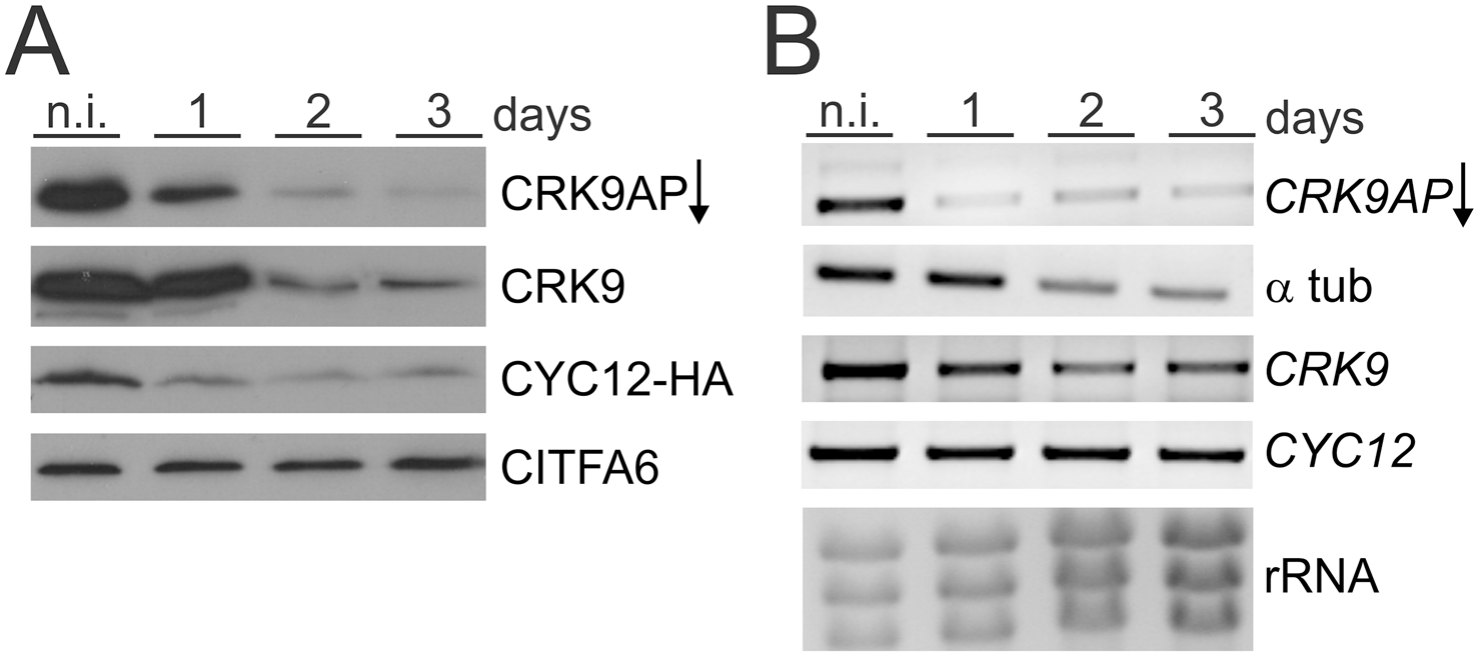
CRK9AP depletion results in rapid co-loss of CRK9 and CYC12. (A) Immunoblot of whole cell lysates derived from non-induced (n.i.) and *CRK9AP*-silenced PF trypanosomes. The arrow indicates the gene knockdown of *CRK9AP*. Detection of the class I transcription factor A subunit 6 (CITFA6) served as a loading control. (B) Corresponding semi-quantitative PCR analysis of cDNA that was obtained from the same cells by reverse transcription of total RNA using oligo-dT. Relative RNA amounts were determined by ethidium bromide–stained rRNA.

A tripartite CRK9 enzyme complex would be highly unusual since, to our knowledge, eukaryotic CDK7 is the only CDK whose enzyme activity depends on such a complex. Thus, to analyze the specific function of CRK9AP, we investigated the effect of CRK9AP ablation on the expression of its functional partners. CRK9 and CYC12 proteins were rapidly lost in PFs upon *CRK9AP* silencing, unlike a control protein (Fig 5A), although their mRNA levels were only moderately affected, most likely due to the SL *trans* splicing block (Fig 5B). A similar co-loss of CRK9 and CYC12 was observed in BFs (S8A Fig). Interestingly, depletion of CRK9 caused a co-loss of CYC12 and *CYC12* silencing led to a reduction in CRK9 abundance whereas CRK9AP levels remained unaffected by both gene knockdowns, indicating that the expression level of CRK9AP, in contrast to that of CRK9 and CYC12, was not dependent on the formation of a CRK9 enzyme complex (S8B and S8C Fig). These results suggested that CRK9AP is important for CRK9 complex assembly and/or integrity. Alternatively, CRK9AP may mediate nuclear import of the complex, although the amino acid sequence does not harbor a recognizable nuclear localization signal.

### Recombinant CRK9, CYC12 and CRK9AP form a tripartite autophosphorylating complex

**Fig 6:**
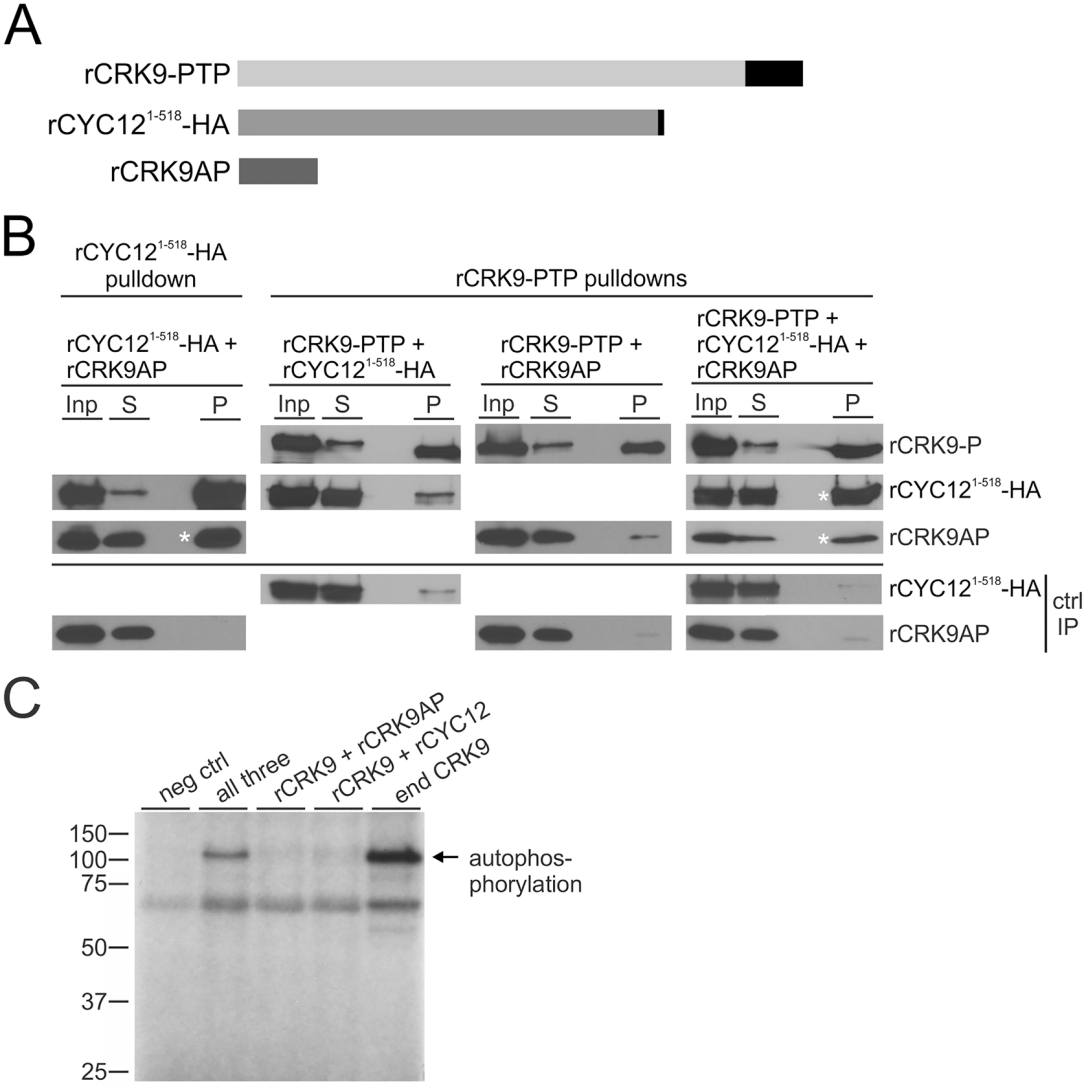
CRK9AP is essential for CRK9 enzyme assembly and autophosphorylation. (A) Schematic to scale of recombinant CRK9-PTP, CYC12^1-518^-HA, and CRK9AP proteins that were expressed in wheat germ extract. PTP and HA tags are depicted as black boxes. (B) rCYC12^1-518^-HA and rCRK9-PTP were pulled down from extract by anti-HA and IgG beads that bind to ProtA of the PTP tag, respectively. Pulldown and co-precipitation (asterisks) of CRK9 complex subunits were analyzed by immunoblotting with anti-ProtC (PTP tag), anti-HA and anti-CRK9AP antibodies, detecting the three proteins in extract (Inp), supernatant (S) and precipitate (P) which was loaded in six-fold excess to extract and supernatant. Negative control pulldowns (ctrl IP) were carried out with extract in which the target protein was not expressed. Note that IgG beads but not anti-HA beads reproducibly co-precipitated minor amounts of either rCYC12^1-518^-HA and rCRK9AP in the control assays. (C) Kinase assay after IgG affinity chromatography and TEV protease release of rCRK9-P in the presence of all three complex components or with either CRK9AP or rCYC12^1-518^-HA. In a negative control (neg ctrl), the assay was carried out without expression of trypanosome proteins and, in a positive control (end CRK9), CRK9 autophosphorylation was achieved by the endogenous CRK9 complex that was tandem affinity-purified from trypanosome extract. The labeled CRK9-P band is indicated on the right (autophosphorylation).

In order to analyze the nature of the CRK9 enzyme complex, we attempted to express recombinant (r) proteins and reconstitute the enzyme complex. In *E*. *coli*, only rGST-CRK9AP was well expressed in soluble form whereas, despite numerous attempts, we failed to express and purify correctly folded GST- and Sumo/6xHis-tagged versions of CRK9 and CYC12. In addition, we realized that full length CYC12 had a strong tendency to aggregate in any form of purification, resulting in protein loss in various assays (f.ex. substoichiometric amounts of CYC12 in the co-sedimenting enzyme complex in Fig 2A). Since aggregation is a common problem of SR and SR-like proteins [51], and the use of arginine and glutamine salts [52] did not decisively improve solubility of CYC12, we attempted to reconstitute the CRK9 enzyme complex in wheat germ extract by co-expressing PTP-tagged CRK9, CRK9AP and a C-terminally HA-tagged CYC12 from which residues 519 to 639, comprising the charged domain, were deleted (CYC12^1-518^-HA) (Fig 6A). We expressed pairs of CRK9 complex subunits and all three proteins together to analyze their interactions by co-IP (Fig 6B). In these assays, rCYC12^1-518^-HA clearly formed a complex with rCRK9AP in the absence of rCRK9-PTP whereas rCRK9-PTP required the expression of both rCYC12^1-518^-HA and rCRK9AP for efficient interaction with these subunits. Thus, it appears that CYC12 and CRK9AP need to interact before they can form a complex with CRK9, strongly indicating that CRK9AP is crucially important for the formation of an active CRK9 complex. This notion was corroborated by conducting a kinase assay with immunoprecipitated rCRK9-PTP (Fig 6C). When the enzyme was expressed with either rCRK9AP or rCYC12^1-518^-HA, we observed very minor labeling of the kinase band whereas autophosphorylation was clearly detectable upon co-expression of all three proteins. Together, these results strongly indicate that CRK9 kinase activity requires formation of a tripartite enzyme complex. It remains to be determined, however, whether CRK9AP is strictly an assembly factor or directly involved in the formation of the active site.

#### Validation of CRK9 as a potential chemotherapeutic target in the mouse model

**Fig 7:**
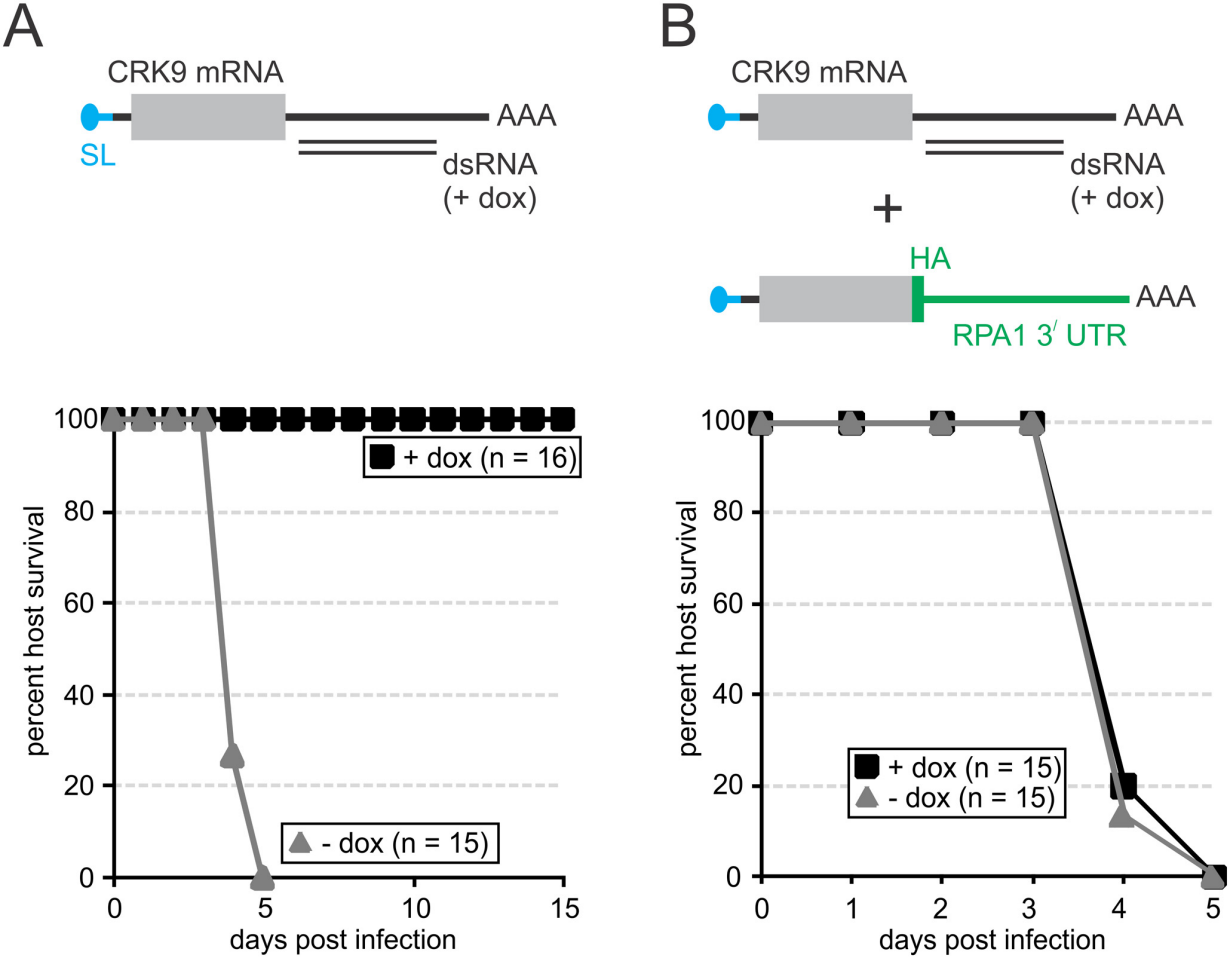
Validation of CRK9 as a drug target in the mouse. (A and B, top) Depiction of CRK9 mRNAs and the targeting dsRNA in two cell lines derived from the *T*. *brucei brucei* 427 smBF cell line which were used for mouse infection studies. The cell line on the left (A) harbored a construct for conditional expression of dsRNA that targets the 3^/^ UTR of the *CRK9* mRNA. This line was further modified (B) by targeted integration of a plasmid into the endogenous *CRK9* locus that fused a functional HA tag sequence and the 3^/^ UTR of *RPA1* to the 3^/^ end of one *CRK9* allele, making the corresponding mRNA resistant to the RNAi response. As the survival graphs of infected mice show in the bottom panels, doxycycline treatment rescued every single mouse when CRK9 was depleted. This effect was completely abolished upon introduction of an RNAi-resistant *CRK9* gene into the same trypanosomes.

The unique nature of the CRK9 enzyme complex, its central importance to trypanosome gene expression, and its membership in the druggable CDK family makes CRK9 a potential chemotherapeutic target for kinetoplastid parasites. Thus, as an important first step in this direction, we validated CRK9 as a drug target in the mouse host. We first used so-called single marker BF (smBF) trypanosomes, the parent line for conditional gene silencing experiments [53], to determine their lethal dose in intraperitoneal BALB/c mouse infections. Injection of 2 million smBFs was lethal to mice within 3–5 days. Accordingly, when mice were infected with a modified smBF cell line, in which doxycycline triggered effective *CRK9* silencing through expression of *CRK9* 3^/^ UTR dsRNA (S9 Fig), mice died on days 4 and 5 in the absence of the compound (n = 15; Fig 7A). Conversely, when mice (n = 16) received doxycycline in their drinking water, they all survived for two weeks, the time doxycycline was administered (Fig 7A). To ensure that this effect was due to CRK9 ablation and not an off-target effect, we introduced a *CRK9* transgene, which was resistant to RNAi due to a different 3^/^ UTR sequence, into the *CRK9* knockdown cell line. As expected, culture growth of these cells was not affected by doxycycline (S9 Fig) and these trypanosomes were lethal to all mice in the absence (n = 15) and presence (n = 15) of doxycycline (Fig 7B and 7C). Finally, since CRK9’s kinase activity depends on CYC12, we wanted to find out whether *CYC12* silencing cures mice infections. Thus, we sacrificed ten more mice by infecting them with the BF *CYC12* knockdown cell line characterized in S6 Fig. While the five doxycyline-treated mice survived for the two week span of the experiment the five untreated mice died on day 5. Thus, we concluded that inhibition of CRK9 kinase activity is a valid strategy to combat trypanosome infections of mammalian hosts.

## Discussion

CRK9 is the first ‘transcriptional’ CDK of trypanosomes that is of crucial importance to the parasite-specific mode of gene expression. Ablation of CRK9 blocked SL *trans* splicing, led to an accumulation of SL RNA with a hypomethylated cap structure and caused a loss of RPB1 phosphorylation [10]. Here we identified two un-annotated proteins that co-purified and co-sedimented with CRK9. Multiple sequence alignment and phylogenetic analysis suggests that the larger protein, CYC12, is an L-type cyclin that has not been recognized before due to unique 74- and 45-aa sequence insertions in its Cyclin_N and Cyclin_C domains, respectively. More support for CYC12 being a cyclin L comes from its domain structure which includes a highly positively charged C-terminal domain that contains a moderate accumulation of SR dipeptides reminiscent of the characteristic C-terminal RS domain of L-type cyclins [24]. Previous pull-down experiments with recombinant CRK9 suggested that CRK9 binds to cyclin CYC6 (aka B2) but not to CYC1 (aka E1) [29]. However, our mass spectrometric analyses of CRK9-copurified proteins did not identify a CYC6-specific peptide or peptides from other annotated cyclins, indicating that CRK9, as is typical for ‘transcriptional’ CDKs, interacts exclusively with CYC12. Cyclins L are known binding partners of CDK11, suggesting that CRK9 is a functional homolog of this kinase. CDK11 is also known as the PITSLRE kinase due to the presence of this motif in its cyclin-binding helix domain [19]. Interestingly, this motif is perfectly conserved in trypanosome CRK12 whereas kinetoplastid CRK9s possess a PPYL/MLRE motif [27]. On the other hand, CRK9 but not CRK12 clustered with the CDK10/CDK11 branch of a phylogenetic tree constructed by eukaryotic CDK sequences with a bootstrap value of 45% [42].

The notion that CRK9 is a functional homolog of CDK11 is strongly supported by functional overlap. Consistent with the SL *trans* splicing block observed upon interference with the CRK9 complex, the CDK11^p110^ isoform of human CDK11 proved to be essential for pre-mRNA splicing both *in vivo* and *in vitro* [22, 24, 54, 55]. Furthermore, CDK11 was co-purified with spliceosomal A and B complexes that form before the first splicing step [56] which agrees with CRK9 being essential for the first transesterification to occur. The spliceosomal SR protein SRSF7 (aka 9G8) was identified as a substrate of CDK11^p110^ [55], and the SR-related splicing factor RNPS1 as a direct interactor of the kinase [57]. Correspondingly, the trypanosome SR proteins TSR1 and branch point binding protein SF1, both of which are essential for SL *trans* splicing [48, 58], co-purified with CRK9 (Tables 1 and S1). Moreover, consistent with the observed RPB1 dephosphorylation upon silencing *CRK9*, *CYC12* and *CRK9AP* genes in trypanosomes, human CDK11 co-purified with RNA pol II complexes and transcription elongation factors [24, 59, 60], indicating that both mammalian CDK11 and trypanosome CRK9 play central roles in the coordination and regulation of transcription and RNA processing. Although the CDK11 homolog in *S*. *pombe* may function in a specific manner in transcription by regulating the assembly of the co-activating mediator complex [61], this finding underscores the evolutionary conserved role of CDK11 in transcription which may be shared by CRK9. Finally, both CRK9 and CDK11 are important for the cell cycle. The human CDK11^p58^ isoform was shown to be important for centriole duplication, centrosome maturation, bipolar spindle assembly, maintenance of sister chromatid cohesion and cytokinesis, leading to the accumulation of binucleate cells [15–18]. Accordingly, CRK9-depleted trypanosomes exhibited a defect in basal body segregation and cells in G2/M phase [29].

While CRK9’s participation in gene expression and the cell cycle, and the identification of an L-type cyclin as its partner strongly argue that it is a functional CDK11 homolog, CRK9, nevertheless, exhibits features that clearly distinguish it from CDK11s of other organisms. As mentioned above, the cyclin-binding helix domain motif PPYL/MLRE and the multiple sequence insertions in both the cyclin domains of CYC12 and the kinase domain of CRK9 [27] are unique. Most strikingly though is the fact that CRK9 forms a tripartite complex with CYC12 and the non-cyclin CRK9AP. We provided several lines of evidence that CRK9AP is an integral part of the enzyme complex: ablation of CRK9AP resulted in the same effects on RNA splicing, RPB1 phosphorylation, and trypanosome morphology as did *CRK9* silencing (Fig 4, S5–S7 Figs), identifying it as a functional CRK9 partner *in vivo*. *CRK9AP* silencing also led to a rapid co-loss of CRK9 and CYC12 protein (Fig 5), suggesting that CRK9AP is important for the integrity of the enzyme complex. Finally, recombinant CRK9 in wheat germ extract interacted with CYC12^1-518^-HA and was active in autophosphorylation only when CRK9AP was co-expressed (Fig 7), validating CRK9AP’s essential function in the formation of an active CRK9 complex. To our knowledge, CDK7 is the only other CDK that forms a *bona fide* tripartite complex with cyclin H and MAT1. This unique parallel suggests that CRK9 and CDK7 may have a common function. Could the CRK9/CYC12/CRK9AP trimeric complex represent a divergent CAK complex? This possibility cannot be ruled out because the phenotype of CRK9 ablation in PFs suggested multiple defects in mitosis and in the segregation of kinetoplasts and basal bodies [29]. On the other hand, conserved from yeast to mammals, CAK activity is necessary for CDK1 activation that is critically important for mitosis [62]. Accordingly, silencing of *CRK3* or *CYC6*, encoding the trypanosome CDK1 ortholog and its mitotic partner cyclin, respectively, caused a mitotic block in BF and PFs. In PFs, these knockdowns did not affect cytokinesis and resulted in the generation of anucleate cells with a single kinetoplast termed zoids [33, 35, 36]. Zoids, however, were not observed in PFs depleted of CRK9, CYC12 or CRK9AP [29] (this study). Additionally, co-purification of other CDKs with CRK9, which could suggest a direct interaction, was not observed.

Interestingly, in *S*. *cerevisiae*, CDK activation is carried out by the single-subunit kinase CAK1 whereas the CDK7 ortholog Kin28, which forms the trimeric complex, is part of the TFIIH complex and phosphorylates the CTD of RPB1 [62], suggesting that the transcriptional function of CDK7/Kin28 is of ancient evolutionary origin and the hallmark of the tripartite kinase complex. Hence, does the tripartite CRK9 phosphorylate the RPB1 CTD despite being an apparent CDK11 homolog? Again, this is a possibility since RPB1 dephosphorylation was observed upon depletion of CRK9 complex components. However, RPB1 and other RNA pol II subunits did not co-purify with CRK9. Moreover, there is convincing evidence that trypanosome TFIIH lacks a kinase component and does not directly interact with CRK9: CRK9 was not discovered in eluates of several independent TFIIH tandem affinity purifications [63–65], the low resolution EM structure of isolated TFIIH complexes lacked the knob-like CAK domain of human TFIIH [64], and CRK9 was not found to occupy the SL RNA gene promoter *in vivo*, the only known *T*. *brucei* promoter that assembles a conventional RNA pol II pre-initiation complex [10]. These data suggest that CRK9’s role in CTD phosphorylation, like that of CDK11, is indirect. Human CDK11^p110^ was shown to interact with casein kinase II, which phosphorylates both the CTD and CDK11, indicating that CDK11 is part of a signaling pathway that coordinates transcription and RNA splicing [60]. The fact that three distinct kinases co-purified with CRK9 with high protein scores (Table 1) suggests that the trypanosome enzyme may similarly be involved in gene expression coordination. On the other hand, the presence of two DYRK kinases among the top scoring CRK9 co-purificants may reflect the fact that human cyclin L is a substrate of the nuclear kinase DYRK1A [66].

Is CRK9AP’s role in the CRK9 complex comparable to that of MAT1 in the CAK complex? MAT1 is important for CAK complex assembly *in vitro* [67, 68] and for the integrity of the complex *in vivo* since in MAT1-deficient cells, the abundances of CDK7 and cyclin H are significantly reduced [69–71]. These functions are congruent with our results on CRK9AP. Furthermore, MAT1 stimulates the kinase activity of CDK7 [70, 72]. Although CRK9AP expression was important for CRK9’s autophosphorylation in wheat germ extract (Fig 7C), it remains to be determined whether CRK9AP functions beyond assembly and integrity of the CRK9 complex. Structurally, MAT1 has an N-terminal RING finger involved in CTD phosphorylation, a central coiled-coil domain mediating the interaction of CAK and TFIIH, and a hydrophobic C-terminal domain for binding to the CAK complex [72]. Conversely, CRK9AP does not possess a RING finger, and coiled-coil domain prediction [73] did not return a convincing score for the presence of this structure in CRK9AP. On the other hand, the C-terminal region of CRK9AP has a similar hydrophobic characteristic as MAT1. However, it will require functional dissection of CRK9AP and/or a structural analysis to determine whether this protein shares functional domains with MAT1. Finally, the Cip/Kip family of CDK inhibitors is another group of small proteins which, in addition to their well-known inhibitory roles, were shown to aid assembly and activation of CDK4/6-cyclin D complexes [74, 75]. However, unlike Cip/Kip proteins, CRK9AP’s sequence conservation among kinetoplastids is not restricted to the N-terminus (S1 Fig) and our results with recombinant proteins suggest that CRK9AP has a fundamentally important function in the CRK9 complex.

In summary, CRK9 assembles into a unique complex together with the L-type cyclin CYC12 and CRK9AP, its enzymatic activity is absolutely required for trypanosome viability and SL *trans* splicing, a kinetoplastid-specific step of gene expression, and *CRK9* silencing impaired trypanosomes from developing lethal infections in the mouse model (Fig 7). These findings and the facts that CDKs are excellent drug targets in general, that interference with human CDK11 was preclinically found to be therapeutically beneficial [76], and that CRK9, CYC12 and CRK9AP are conserved among all kinetoplastids, identify CRK9 kinase activity as a prime target for combating diseases caused by kinetoplastid parasites. Thus, in the next step, it will be important to identify substrates and signaling pathways as well as establish a high throughput screening assay for this unique kinase.

## Methods

### DNAs

For gene silencing of *CYC12* and *CRK9AP*, their coding regions from position 21 to position 520 and from position 1 to position 414, were integrated in stem-loop arrangements into the pT7-stl vector [77] to obtain plasmids T7-CYC12-stl and T7-CRK9AP-stl, respectively. To target the 3^/^ untranslated region (3′ UTR) of CRK9 for gene silencing, 501 bp of the 3^/^ UTR sequence, from positions 2326 to 2826 relative to the translation initiation codon, was inserted into pT7-stl to generate pT7-CRK9UTR-stl. For HA-tagging of CYC12, 1140 bp of the 3^/^-terminal CYC12 coding sequence was inserted into the pC-HA-BLA vector [78], utilizing the vector's ApaI and NotI restriction sites. The resulting vector, named pCYC12-HA-BLA, was linearized with BamHI for transfection. For expression of N-terminal glutathione *S*-transferase (GST) fusion proteins in *E*. *coli*, the coding regions of CRK9AP and of amino acids 100–300 of CRK9 were cloned into the pGEX-4T-2 vector (GE Healthcare) using the vector’s EcoRI and NotI restriction sites. For expressing CRK9-PTP, CYC12^1-518^-HA and CRK9AP in wheat germ extract, the complete coding sequences of these proteins were cloned into the pSP64 Poly(A) expression vector (Promega) using the vector’s XbaI/SmaI, XbaI/SacI, and HindIII/XbaI restriction sites to yield pSP64-CRK9-PTP, pSP64-CYC12^1-518^-HA and pSP64-CRK9AP, respectively.

DNA oligonucleotides that were used in semi-quantitative and quantitative reverse transcription (RT)-PCR and in primer extension assays are specified in S2 Table.

### Cells

PF and BF *Trypanosoma brucei* cell culture was maintained, transfected, and cloned by limiting dilution as described previously [78, 79]. For conditional *CYC12* and *CRK9AP* silencing experiments linearized pT7-CYC12-stl and pT7-CRK9AP-stl constructs were transfected into PF 29–13 and smBF cells, respectively [53], for targeted integration into the *RRNA* locus. For conditional *CRK9* silencing in BFs, pT7-CRK9UTR-stl, linearized with EcoRV, was transfected into smBF cells. The cell line was further modified by targeted insertion of pCRK9-HA-BLA to generate the rescue cell line. For induction of gene silencing, trypanosomes were incubated in medium containing 2 μg/ml of doxycycline that triggered dsRNA synthesis. Cells were counted and diluted daily to 2×10^6^ cells/ml for PF culture and to 2×10^5^ cells/ml for BF culture. For detection of CYC12 in CRK9 and CRK9AP RNAi cell lines, linearized pCYC12-HA-BLA was transfected and targeted to an endogenous *CYC1*2 allele, fusing the HA tag sequence to the 3^/^ end of the CYC12 coding region. It should be noted that we were unable to knock out the remaining wild-type allele which indicates that deleting a *CYC1*2 allele leads to haplo-insufficiency or that the HA tag partially interfered with CYC12 function.

### RNA analysis

Semi-quantitative and quantitative RT-PCR was performed to determine relative amounts of various RNAs during gene silencing experiments. Total RNA was prepared from 8 x 10^7^ PF or 1 x 10^8^ BF cells using the TRIzol reagent (Invitrogen) or the hot-phenol method [80], respectively. Reverse transcription reactions were carried out with SuperScript II reverse transcriptase (Invitrogen) according to the manufacturer’s specifications, using oligo-dT and random hexanucleotides (Roche) for the analysis of mature, spliced mRNAs and unspliced pre-mRNA, respectively. For each semi-quantitative PCR, the number of cycles for the linear amplification range was determined empirically. For RNA quantifications, cDNA preparations were analyzed by qPCR assays using the SsoFast EvaGreen Supermix (BioRad) on a CFX96 cycler (BioRad) according to the manufacturer’s recommendations. For each amplification, triplicate qPCR samples were analyzed. Oligonucleotide pairs that were used in qPCR reactions were evaluated for specificity by both agarose gel electrophoresis and melting curve analysis. Standard curves for oligonucleotide pairs were obtained from serial dilutions of non-induced cDNA samples and ranged in their coefficient of determination (R^2^) value from 0.98 to 1.0. Samples were standardized with 18S *RRNA* amplification of random hexamer-derived cDNA. The methylation status of SL RNA cap4 was analyzed by a modified primer extension assay as described previously [10].

### Protein analysis

The polyclonal anti-CRK9 and anti-CRK9AP immune sera were generated by immunization of rats [81] with GST-CRK9AP or GST-CRK9^100-300^. The recombinant proteins were expressed in *E*. *coli* strain BL21Star (DE3) and purified by glutathione affinity chromatography (GE Healthcare). The CRK9 polypeptide was chosen due to its hydrophilic nature as determined by a hydrophilicity blot using the Kyte & Doolittle algorithm with default parameters on the ProtScale server at http://web.expasy.org/protscale/.

CRK9 and CRK9AP were detected on immunoblots by 1:1000 dilution of the respective immune sera followed by a 1:5000 dilution of a monoclonal, peroxidase-labeled anti-rat IgG secondary antibody (Vector Laboratories). HA-tagged protein was detected with a commercial monoclonal rat anti-HA antibody (Roche), RPA1 with a polyclonal rabbit immune serum [82] and RPB1 [10], TFIIB [81] and CITFA6 [83] with a polyclonal rat immune serum. Blots were developed with BM chemiluminescence blotting substrate (Roche) according to the manufacturer's protocol. PTP-tagged proteins were detected either with the peroxidase anti-peroxidase reagent (Sigma) or the monoclonal anti-ProtC antibody HPC4 (Roche).

Extract preparation and tandem affinity purification of PTP-tagged CRK9 was carried out according to the standard PTP purification protocol [44]. Purified proteins were separated on SDS–10 to 20% polyacrylamide gradient gels (BioRad) and stained either with SYPRO Ruby (BioRad) or with Coomassie blue (Gelcode Coomassie stain; Thermo Fisher Scientific). For the sedimentation analysis of the CRK9 complex, the final eluate of a standard CRK9-PTP purification was concentrated, dialyzed against E-80 buffer and sedimented in a 4 ml, linear 10–40% sucrose gradient as described before [43]. Briefly, the gradient was fractionated from top to bottom in twenty aliquots of 200 μl each. Proteins from 150 μl of each fraction were collected by a hydrophobic resin (StrataClean, Stratagene), and resuspended in SDS loading buffer for electrophoresis. The remaining 50 μl aliquots were dialyzed against E-20 buffer (20 mM HEPES-KOH pH 7.7, 20 mM potassium glutamate, 20 mM potassium chloride, 3 mM magnesium chloride, 0.2 mM EDTA, 0.5 mM EGTA and 4 mM DTT) and used for *in vitro* kinase assays. Proteins that co-purified with CRK9-P were analyzed in two independent experiments by LC/MS/MS from the gel lane of the final eluate by the Keck Biotechnology Resource Laboratory of Yale University as recently described [43]. Individual protein bands obtained after sucrose gradient sedimentation were analyzed equivalently.

For recombinant protein analysis, proteins were expressed in the TNT SP6 High Yield Wheat Germ Protein Expression System (Promega) according to the manufacturer’s specifications. Briefly, 50 μl reactions containing 3 μg of pSP64-CRK9-PTP, 3 μg of pSP64-CYC12^1-518^-HA and/or 2 μg of pSP64-CRK9AP were incubated for 2 h at 25°C. After pre-clearing the extract for 10 min at 25,000 g and 4°C, 42 μl of the reaction was combined with 25 μl settled volume of IgG Sepharose 6 Fast Flow matrix (GE Healthcare), equilibrated in IP_400_ buffer (400 mM NaCl, 20 mM Tris-HCl, pH 8.0, 3 mM MgCl_2_, 0.1% NP40, 0.5x EDTA-free cOmplete protease inhibitor cocktail [Roche]), and incubated on ice for 1 h. Beads were washed seven times with 0.9 ml of IP400 buffer and immobilized CRK9-PTP was released by TEV protease cleavage at 28°C for 30 min in a 50 μl-reaction containing 150 mM NaCl, 20 mM Tris-HCl, pH 7.7, 0.5 mM EDTA, 1 mM DTT, 0.1% Tween20, and 20 units of TEV protease (Invitrogen). For the kinase assay, 8 μl of the eluate were used in a 40 μl kinase reaction that was incubated at 37°C for 30 min and contained 60 mM KCl, 40 mM Tris-HCl, pH 7.7, 14 mM MgCl_2_, 1 mM DTT, 0.2 mg/ml; BSA, 1 μM ATP, and 57.5 nM [γ-^32^P]ATP (7000 Ci/mmol).

### Phylogenetic analysis

Amino acid sequences spanning the CCL1 domain (COG5333) of human cyclins and of CYC12 from *T*. *brucei*, *Trypanosoma cruzi*, *Leishmania major* and *Bodo saltans* were aligned using the multiple sequence alignment tool ClustalΩ at the EMBL European Bioinformatics Institute (http://www.ebi.ac.uk/Tools/msa/muscle/). The alignment was uploaded onto the graphical user interface Seaview [84] at http://pbil.univ-lyon1.fr/software/seaview.html and a maximum likelihood tree was generated employing the LG empirical matrix [85] with optimized invariable sites, substitution rate categories of 4, estimated gamma distribution and model equilibrium frequencies. Bootstrapping was performed with 1000 replicates.

### Mouse infections

6–10 weeks old female BALB/c mice (The Jackson Laboratory, Bar Harbor, ME), were injected intraperitoneally with 2 × 10^6^ BF trypanosomes that were suspended in 0.1 ml of cold bicine-buffered saline glucose (50mM bicine-NaOH, pH8.0, 50 mM NaCl, 5 mM KCl, 77 mM glucose). To ensure cell viability, the parasites and syringes were kept on ice prior to injections. To induce gene silencing in transfected trypanosomes, mice were given 1 mg / ml doxycycline in their drinking water 2 days prior to injection and for up to 2 weeks post-injection. Drinking water was replaced daily with fresh doxycycline solution. Mice were monitored daily and sacrificed upon signs of distress. Blood samples, randomly taken from sick mice, were investigated microscopically, revealing, in each case, parasitaemias ranging from 0.8 to 1 x 10^9^ trypanosomes per ml of blood.

### Ethics statement

The generation of immune sera in rats and the mouse infection studies were carried out according to protocols which were approved by the UConn Health Institutional Animal Care and Use Committee (Public Health Service [PHS] assurance number A3471-01) and were in accordance with the PHS *Policy for the Humane Care and Use of Vertebrate Animals* and the *Guide for the Care and Use of Laboratory Animals*.

## Supporting Information

S1 Fig CRK9AP is conserved among kinetoplastid organisms. Kinetoplastid CRK9AP sequences were aligned using the Clustal Omega server of the European Bioinformatics Institute (http://www.ebi.ac.uk/Tools/services/web/toolform.ebi?tool=clustalo) at default parameters [1]. Positions with more than 50% identity or similarity are highlighted in black or gray, respectively. Dashes indicate that a corresponding residue is missing. Sequences were obtained from the TriTrypDB (www.TriTrypDB.org [2] or www.GeneDB.org [3]) and comprise those of *T*. *brucei brucei* strains 427 (Tb427, accession number Tb427.03.4170) and 927 (Tb927, Tb927.3.4170), *Trypanosoma vivax* (Tv, TvY486_0303410), *Trypanosoma cruzi* CL Brener Esmeraldo-like (Tc-el; TcCLB.509669.80) and Non-Esmeraldo-like (Tc-nel; TcCLB.506175.30), *Trypanosoma grayi* (Tgr, Tgr.163.1040), *Crithidia fasciculata* (Cfa, CfaC1_25_1880), *Leishmania braziliensis* (Lbr, LbrM.29.1690), *Leishmania tarentolae* (Lta, LtaP29.1740), *Leishmania mexicana* (Lmx, LmxM.08_29.1585), *Leishmania major* (Lm, LmjF.29.1585), *Leishmania infantum* (Li, LinJ.29.1710), *Leishmania donovani* (Ld, LdBPK_291710.1), and the bodonid *Bodo saltans* (Bs, BS14910.1.pep). Please note that the open reading frame of the *T*. *brucei* gene starts at an ATG upstream of the annotated start codon [4].
(DOCX)

S2 Fig Generation of a highly specific rat anti-CRK9AP immune serum. CRK9AP was expressed in *Escherichia coli* as a C-terminal fusion to glutathione S-transferase and purified from bacterial extract by glutathione affinity chromatography. By injecting the purified protein into the rat bloodstream, [pre-]immune serum was obtained according to a published protocol [5]. Pre-immune (pre-IS) and α-CRK9AP immune sera (IS) were used to probe whole cell lysates (wcl) and crude extract (extr) of procyclic *Trypanosoma brucei brucei* strain 427. As a loading control, transcription factor TFIIB was detected on the same blots. Marker sizes in kDa are indicated on the left.
(TIF)

S3 Fig Multiple sequence alignment of the CCL1 domain of eukaryotic cyclin L and kinetoplastid CYC12 proteins. Cyclin L and CYC12 sequences were aligned using the Clustal Omega server at default parameters. Shown are the sequences of the CCL1 domain (COG5333) as defined in human cyclin L1 according to the *Conserved Domain Database* [6]. Dashes indicate that a corresponding residue is missing. Numbers in parentheses specify number of residues without significant sequence similarity. Positions with more than 50% identity or similarity are highlighted in black or gray, respectively. Identical positions in model organisms without similarity in kinetoplastids were highlighted in blue and insertions or unique identical positions in kinetoplastids were highlighted in red. Stars and colons denote positions that are identical or similar in all sequences analyzed. Yellow highlighting indicates cyclin folds 1 and 2 within the CCL1 domain and question marks indicate positions of the human cyclin L1 sequence that were not recognized as part of the CCL1 domain. Cyclin L sequences are from *Homo sapiens* (*Hs*; L1, accession number NP_064703; L2A, NP_112199), *Mus musculus* (*Mm*, NP_064321), *Danio rerio* (*Dr*, NP_956034), *Caenorhabditis elegans* (*Ce*, NP_506007), *Arabidopsis thaliana* (*At*, NP_565622) and *Schizosaccharomyces pombe* (*Sp*, NP_593045). Kinetoplastid CYC12 sequences were from *T*. *brucei* (*Tb*, accession number Tb927.10.9160), *Trypanosoma cruzi* (*Tc*, TcCLB.503525.20), *Leishmania major* (*Lm*, LmjF.36.5640), and the bodonid *Bodo saltans* (*Bs*, BS70770.1).
(DOCX)

S4 Fig CYC12 clusters phylogenetically with cyclin L of model organisms. Amino acid sequences of cyclins from *H*. *sapiens* (Hs), *M*. *musculus* (Mm), *Drosophila melanogaster* (Dm), *C*. *elegans* (Ce), *A*. *thaliana* (At), *S*. *cerevisiae* (Sc), and *S*. *pombe* (Sp) as well as kinetoplastid CYC12 sequences from *Trypanosoma brucei brucei* strains 427 (Tb427) and 927 (Tb927), *Trypanosoma congolense* (Tco), *Trypanosoma vivax* (Tv) *T*. *cruzi* (Tc), *L*. *major* (Lm), *Leishmania infantum* (Li), *Leishmania donovani* (Ldon), *Leishmania mexicana* (Lmex), *Leishmania tarentolae* (Ltar), *Leishmania braziliensis* (Lbr), and the bodonid *B*. *saltans* (Bs) were aligned using the Clustal Omega server at http://www.ebi.ac.uk [7]. The multiple sequence alignment was imported into the ClustalX software package [8] and phylogenetically analyzed using the neighborhood joining method. Bootstrap values were obtained by sampling a thousand replicates and are indicated as percentages. The node for the cyclin L/CYC12 cluster is drawn in red. Cyclin clusters of cell cycle-regulating and transcriptional CDKs, according to Ma *et al*. [9], are indicated. The cyclin sequences were obtained from the following accession numbers: Hs_A (CAA35986.1), Hs_B1 (CAO99273.1), Hs_B2 (AAI05087.1), Hs_B3 (CAC94915.1), Hs_C (AAH41123.1), Hs_D1 (AAH23620.1), Hs_D2 (CAA48493.1), Hs_D3 (AAA52137.1), Hs_E1 (AAH35498.1), Hs_E2 (AAC78145.1), Hs_F (AAB60342.1), Hs_G1 (AAC78145.1), Hs_G2 (AAC41978.1), Hs_H (AAA57006.1), Hs_I (AAF43786.1), Hs_J (AAH43175.1), Hs_K (AAH43175.1), Hs_L1 (AAH43175.1), Hs_L2A (Q96S94.1), Hs_O (NP_066970.3), Hs_T1 (AAC39664.1), Hs_T2 (AAW56073.1), Hs_Y (AAH94815.1), Mm_A (CAA81331.1), Mm_B1 (AAH85238.1), Mm_B2 (AAH08247.1), Mm_B3 (AAI38356.1), Mm_C (AAH03344.2), Mm_D1 (AAO13813.1), Mm_D2 (AAH49086.1), Mm_D3 (AAC53363.1), Mm_E1 (AAI38663.1), Mm_E1 (AAC80527.1) Mm_F (AAA63152.1), Mm_G1 (AAC42082.1), Mm_G2 (AAC32372.1), Mm_H (AAH38861.1), Mm_I (AAF43391.1), Mm_J (AAI20923.1), Mm_K (AAH27297.1), Mm_L1 (AAH94383.1), Mm_L2 (AAI32296.1), Mm_O (AAI47760.1), Mm_T1 (AAD13656.1), Mm_T2 (AAH54122.1), Mm_Y (NP_080760.2), Dm_A (NP_524030.2), Dm_B (AAF46904.1), Dm_C (CAA44720.1) Dm_D (NP_523355.2), Dm_E (AAF53477.1), Dm_H (NP_524207.1), Dm_K (AAN11146.1), Dm_L (putative, Dm_T (AAS64974.1), Dm_Y (AAF53122.1), Ce_A (AAA84393.1), Ce_B1 (Q10653.1), Ce_B3 (AAA84395.1), Ce_C (Q9TYP2.2), Ce_D (AAC35273.1), Ce_E (AAM78547.1), Ce_H (NP_494564.2), Ce_L (AAS64750.1), Ce_T1.1 (P34425.1), Ce_T1.2 (P34424.2), Ce_Y1 (NP_498857.2), Ce_Y2 (NP_498858.2), At_A1 (Q9C6Y3.1), At_A2 (AED93433.1), At_A3 (NP_199122.1), At_B1 (P30183.2), At_B2 (Q39068.2), At_B3 (NP_173083.3), At_D1 (P42751.3), At_D2 (P42752.3), At_D3 (P42753.3), At_H (BAB72144.1), At_L (Q8RWV3.2), At_T1 (NP_174775.1), Sc_CLB1 (CAA97112.1), Sc_CLB2 (CAA44195.1), Sc_CLB3 (CAA49201.1), Sc_CLB4 (CAA49202.1), Sc_CLB5 (AAA34503.1), Sc_6 (NP_011623.3), Sc_CLN1 (NP_013926.1), Sc_CLN2 (CAA97982.1) Sc_CLN3 (NP_009360.1), Sp_C (NP_595953.1), Sp_H (NP_595776.1), Sp_L (NP_593045.1), Sp_T (NP_596149.1) Sp_Puc1 (NP_596539.1), Sp_Btype1 (NP_588110.2), Sp_Btype2 (NP_595171.1), Sp_Btype3 (NP_593889.1), Tb927_CYC12 (Tb927.10.9160), Tb427_CYC12 (Tb427.10.9160), Tc_CYC12 (TcCLB.503525.20), Tv_CYC12 (TvY486_1009010), Tco_CYC12 (TcIL3000_10_7930), Lm_CYC12 (LmjF.36.5640), Ltar_CYC12 (LtaP36.5790), Lmex_CYC12 (LmxM.36.5640), Ldon_CYC12 (LdBPK_365890.1), Li_CYC12 (LinJ.36.5890), Lbr_CYC12 (LbrM.35.5920) and Bs_CYC12 (BS70770.1).
(TIF)

S5 Fig *CYC12* and *CRK9AP* silencing. Growth curves of additional PF and BF cell lines in which either *CYC12* or *CRK9AP* was conditionally silenced by doxycycline (dox)-induced dsRNA synthesis. The cross indicates that no viable cells were detectable after day 3 of induction. Corresponding cell lines were analyzed in detail as shown in Fig 4 and S6 Fig.
(TIF)

S6 Fig *CYC12* and *CRK9AP* silencing in BFs. (A) Growth curves of uninduced (- dox) BF cultures or cultures in which *CYC12* or *CRK9AP* was conditionally silenced by the addition of doxycyline to the medium (+ dox). The cross indicates that subsequent to day 3 of induction no intact cells were detectable by microscopic inspection. (B) RNA analyses. Total RNA was prepared from [un-]induced cells and the relative amounts of mature CYC12/CRK9AP and α tubulin mRNA was determined by reverse transcription using an oligo-dT primer and semi-quantitative PCR, performed in the linear range of the amplification reaction. Unspliced, α tubulin pre-mRNA was analyzed by reverse transcription of the same total RNA preparations using random hexamers and PCR with an oligonucleotide that hybridized upstream of the α tubulin SL addition site. rRNA served as a loading control and was detected after RNA separation on agarose gel by ethidium bromide staining. (C) *CRK9* silencing in BFs causing a SL *trans* splicing defect was published previously [10]. Here, immunoblotting of whole cell lysates from these cells shows that RPB1 phosphorylation was lost after one day of induction (unphos.). Corresponding results were obtained when *CYC12* or *CRK9AP* was silenced, although RPB1 dephosphorylation in *CRK9AP*-silenced cells was consistently observable only after two days of induction.
(TIF)

S7 Fig Depletion of CRK9, CYC12 or CRK9AP leads to round-up of cells. Microscopic images of un-induced procyclic cells that look normal (top row) and of rounded cells from the same cell lines (bottom row) when CRK9, CYC12 or CRK9AP was depleted for three days. White scale bars correspond to 10 μm.
(TIF)

S8 Fig CRK9 and CYC12 but not CRK9AP are lost upon interference with the CRK9 enzyme complex in BFs. Immunoblotting of whole cell lysates prepared from BFs in which *CRK9AP* (A), *CRK9* (B) or *CYC12* (C) were silenced, detecting CRK9, CRK9AP and, as a loading control, the transcription factor subunit CITFA6 with specific polyclonal immune sera and CYC12-HA with a monoclonal anti-HA antibody. Note that silencing specificity and efficiency for the *CYC12* RNAi cell line, which harbored immunologically undetectable, endogenous CYC12, was demonstrated on the RNA level in S6 Fig. The protein profile of silenced genes is indicated by an arrow. n.i., non-induced. The experiment shows that CYC12 and CRK9 are lost when other CRK9 complex subunits are depleted whereas CRK9AP abundance remained unaffected and only diminished when the *CRK9AP* gene was silenced.
(TIF)

S9 Fig Analysis of *CRK9* silencing in cultured BF cell lines used in mouse infections. (A and B, top) Schematics reproduced from Fig 7, depicting targeting and rescue of *CRK9* mRNA in the two smBF cell lines that were used in mouse infections studies. Culture growth curves on the bottom are from non-induced cells (- dox) and doxycycline-induced (+ dox) trypanosomes of the corresponding cell lines. (C and D) Semi-quantitative RT-PCR analysis of *CRK9* mRNA in total RNA preparations from non-induced (n.i.) and one day-induced cells, demonstrating efficient *CRK9* silencing. rRNA, visualized by ethidium bromide staining, served as a control for RNA input. (E) Anti-HA immunoblot showed that CRK9-HA protein, expressed from the RNAi-resistant transgene, was unaffected by addition of doxycycline. TFIIB was probed on the same blot as a loading control.
(TIF)

S1 Table CRK9-PTP co-purified proteins. List of proteins that were identified in two CRK9-PTP tandem affinity purifications by LC/MS/MS. The list shows the protein IDs of the second, more comprehensive mass spectrometric analysis. All protein identifications with an Expect value smaller than 0.001 are listed and ranked according to their Mascot Scores. Proteins that were also identified in the first analysis are marked with an asterisk after their accession numbers. Peptides were identified in both the *T*. *brucei brucei* 927 and 427 genome databases at www.TriTrypDB.org. Annotations are according to the 927 database. Top-scoring and “hypothetical, conserved” proteins were analyzed by standard BLASTP searches of the Human Genome database. CRK9 complex subunits are highlighted in yellow, ribosomal proteins in green, non-ribosomal proteins in orange, and standard PTP purification contaminants in gray.
(XLSX)

S2 Table List of oligonucleotides used in RNA analysis.</ SI_Caption>
(DOCX)

S1 References Supporting References. (DOCX)
