# Supplementary material for: Cyclin-Dependent Kinase CRK9, Required for Spliced Leader trans Splicing of Pre-mRNA in Trypanosomes, Functions in a Complex with a New L-Type Cyclin and a Kinetoplastid-Specific Protein
Source: PLoS Pathog. 2016 Mar 8;12(3):e1005498. doi: 10.1371/journal.ppat.1005498 (PMC4783070; doi:10.1371/journal.ppat.1005498)
Supplement: S1 Fig — Kinetoplastid CRK9AP sequences were aligned using the Clustal Omega server of the European Bioinformatics Institute (http://www.ebi.ac.uk/Tools/services/web/toolform.ebi?tool=clustalo) at default parameters [1]. Positions with more than 50% identity or similarity are highlighted in black or gray, respectively. Dashes indicate that a corresponding residue is missing. Sequences were obtained from the TriTrypDB (www.TriTrypDB.org [2] or www.GeneDB.org [3]) and comprise those of T. brucei brucei strains 427 (Tb427, accession number Tb427.03.4170) and 927 (Tb927, Tb927.3.4170), Trypanosoma vivax (Tv, TvY486_0303410), Trypanosoma cruzi CL Brener Esmeraldo-like (Tc-el; TcCLB.509669.80) and Non-Esmeraldo-like (Tc-nel; TcCLB.506175.30), Trypanosoma grayi (Tgr, Tgr.163.1040), Crithidia fasciculata (Cfa, CfaC1_25_1880), Leishmania braziliensis (Lbr, LbrM.29.1690), Leishmania tarentolae (Lta, LtaP29.1740), Leishmania mexicana (Lmx, LmxM.08_29.1585), Leishmania major (Lm, LmjF.29.1585), Leishmania infantum (Li, LinJ.29.1710), Leishmania donovani (Ld, LdBPK_291710.1), and the bodonid Bodo saltans (Bs, BS14910.1.pep). Please note that the open reading frame of the T. brucei gene starts at an ATG upstream of the annotated start codon [4]. (DOCX) [file ppat.1005498.s001.docx]

**Tb427 ----------MSSNNPSDIRIRTELLQEMQLLHAQQEGMKNIRQSEASHSDENDKKERGT 50**

**Tb927 ----------MSSNNPSDIRIRTELLQEMQLLHAQQEGMKNIRQSEASHSDENDKKERGT 50**

**Tv ----------MPAVDPLQV--QTELLERMKTLHVQHMQSLGRRTVNKGS--GASNGVKGT 46**

**Tc-el MDLPPSERPAMPESGPLAFRARTVLLEQLRDLHRQEQQQKKRRGQLRPSNNGPQTGT--- 57**

**Tc-nel MDLPPSENPALSESGPLAFRARTALLEQLRDLHRQEQQQQKRRGQLRLSNNGPQTGT--- 57**

**Tgr ----------MSENVSSKFCARTALLQQLLETC--EEQQKQQRAVQRWSRPEAVSGERTE 48**

**Cfa ----MSSDPNNGTSAGGALRLRTSLLEAVERYNTAQLASAAASDK-----N--------- 42**

**Lbr ---MQSRKDTPTRHTGGTLRIRTALLESVCHYNEAYLSTLTAAAA-----MEEPLET--- 49**

**Lta ---MPGGKDTTTTGRGGRLQIRTALLESVRRYNEDCLNTSTATAV-----VEEPLGT--- 49**

**Lmx ---MQGTKGAPTTNSGGKLQIRTALLESVRRYNEECLNMSTATTA-----AEEPLAT--- 49**

**Lm ---MQGAKDTPTANSGGKLQIRTTLLEAVRRYNEDCLNTSTAAAA-----AEEPLAT--- 49**

**Li ---MRGTNDTPTANSGGKLQIRTALLESVRRYNEDCLNTSTAA-A-----AEEPLAT--- 48**

**Ld ---MRGTNDTPTANSGGKLQIRTALLESVRRYNEDCLNTSTAA-A-----AEEPLAT--- 48**

**Bs ------------------------MMQELRQQQQQQQQSVGSTSSSRLT----------- 25**

**Tb427 RSGSTTGTCSEFDVCAVYDDDDVPLAAQFVRSCPPLTDPSWAMEELLRINSHVAVWT-GS 109**

**Tb927 RSGSTTGTCSEFDVCAVYDDDDVPLAAQFVRSCPPLTDPSWAMEELLRINSHVAVWT-GS 109**

**Tv VAHGSD-GGKDMVNCEGIREDDLPLAVQFVRSCPPLTNPTWAMEELLRINPNVAVWT-GS 104**

**Tc-el ESN-------NDDDSDDESDNSAPMAVRFVRGCPPLTDPSWAMEELLRIDPNVALWT-GT 109**

**Tc-nel ESN-------NDDDSDDESDNSAPMAVRFVRGCPPLTDPSWAMEELLRIDPNVALWT-GT 109**

**Tgr AVH---TAKSDSEDDGEESDNNVSTAVWLMRNCAPLTNPSWAMEELQRIHRGTPVWAAGT 105**

**Cfa ---------------DGEFS---SLVKVLVQRAPNVTNPSWAMEECGRLCPSAELWS-GS 83**

**Lbr ----------SKQSVDRFPD---VLPRMIAQLNPSLTNPSWAMEECMRLCPSAEVWC-GT 95**

**Lta ----------PTQRVDKFPD---ALPRMIAQLYPSLTNPSWAMEECVRLCPSAEVWC-GT 95**

**Lmx ----------QTQRIDRFPD---ALPRMIAQLYPSLTNPSWAMEECVRLCPAAEVWC-GT 95**

**Lm ----------PTQRVDRFPD---ALPRMIAQLYPSLTNPSWAMEECVRLCPAAEVWC-GT 95**

**Li ----------PTQRGDRFPD---ALPRMIAQLYPSLTNPSWAMEECVRLCPAAEVWC-GT 94**

**Ld ----------PTQRGDRFPD---ALPRMIAQLYPSLTNPSWAMEECVRLCPAAEVWC-GT 94**

**Bs -------------------S---SEITDAIRQDEEMSHPNWTHEEMARTNKACPSGG-DS 62**

**Tb427 ALPNSVWALPGS---------GHCPFGSTDADFARIR 137**

**Tb927 ALPNSVWALPGS---------GHCPFGSTDADFARIR 137**

**Tv TLSSTVWAPPAS---------GHCPFGTTSGDFVRHR 132**

**Tc-el GLSTAVWAPPGS---------GHCPFGTTQADFIRHR 137**

**Tc-nel GLSTAVWAPPGS---------GHCPFGTTQADFIRHR 137**

**Tgr QLSSAVWAPPGT---------ANCPFGTKPTDFLRHS 133**

**Cfa ALPLDVWAPPGT---------PDCPFGTTPADFRVHH 111**

**Lbr ALPKDVWAPPGT---------AECPFGTEPMDFNAHR 123**

**Lta ALSKDVWAPPGT---------AECPFGTEPMDFNAHR 123**

**Lmx ALPKDVWAPPGT---------AECPFGTEPMDFNAHR 123**

**Lm ALPKDVWAPPGT---------AECPFGTEPMDFNAHR 123**

**Li ALPKDVWAPPGT---------AECPFGTEPMDFNAHR 122**

**Ld ALPKDVWAPPGT---------AECPFGTEPMDFNAHR 122**

**Bs RLMSTVWTPPRRQPPAAHLPSFPCPFNLQYQDVISVQHRSH 103**
