# Supplementary figures and images for: Cyclin-Dependent Kinase CRK9, Required for Spliced Leader trans Splicing of Pre-mRNA in Trypanosomes, Functions in a Complex with a New L-Type Cyclin and a Kinetoplastid-Specific Protein
Source: PLoS Pathog. 2016 Mar 8;12(3):e1005498. doi: 10.1371/journal.ppat.1005498 (PMC4783070; doi:10.1371/journal.ppat.1005498)

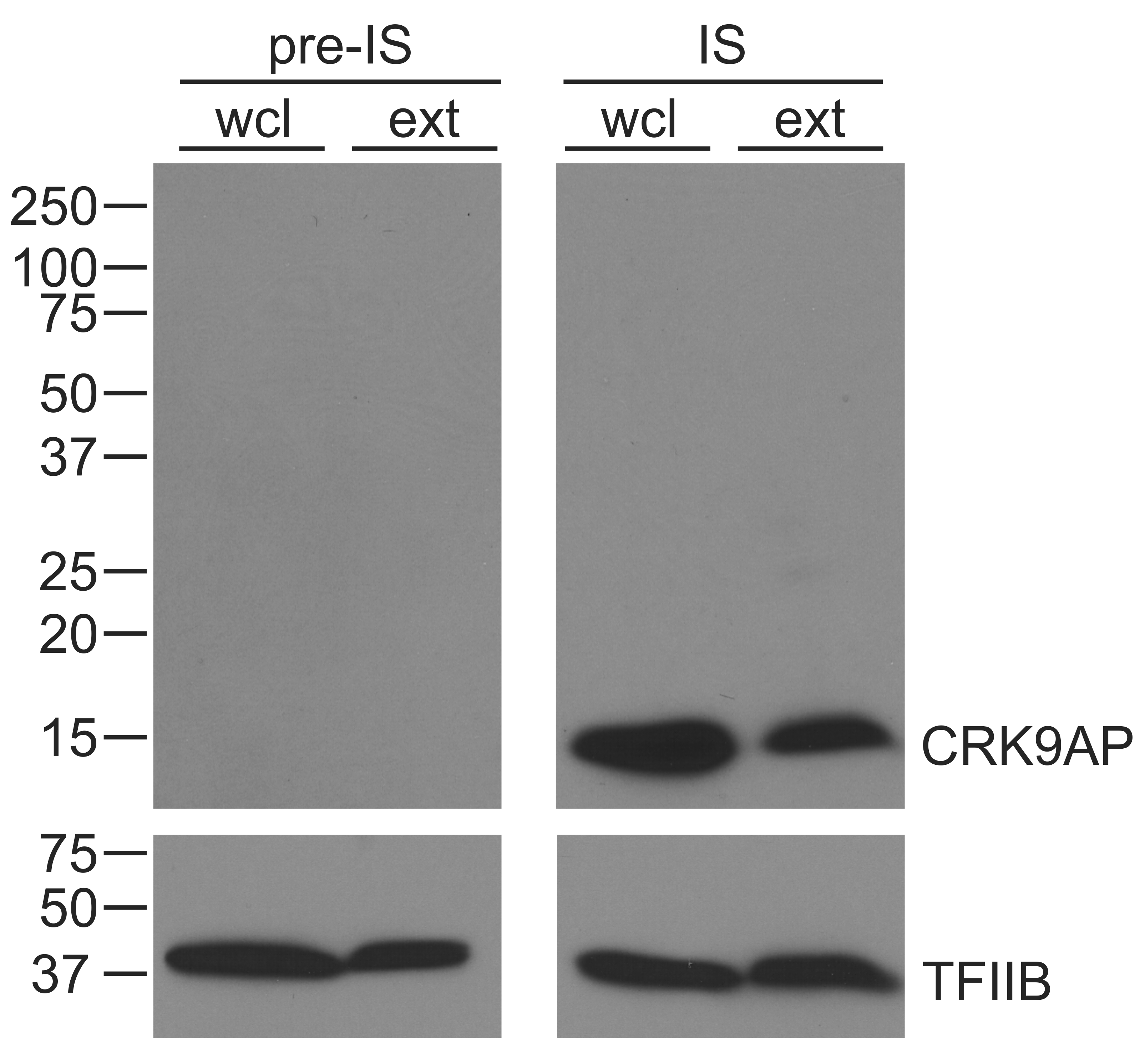

Supplement: S2 Fig — CRK9AP was expressed in Escherichia coli as a C-terminal fusion to glutathione S-transferase and purified from bacterial extract by glutathione affinity chromatography. By injecting the purified protein into the rat bloodstream, [pre-]immune serum was obtained according to a published protocol [5]. Pre-immune (pre-IS) and α-CRK9AP immune sera (IS) were used to probe whole cell lysates (wcl) and crude extract (extr) of procyclic Trypanosoma brucei brucei strain 427. As a loading control, transcription factor TFIIB was detected on the same blots. Marker sizes in kDa are indicated on the left. (TIF) [file ppat.1005498.s002.tif]

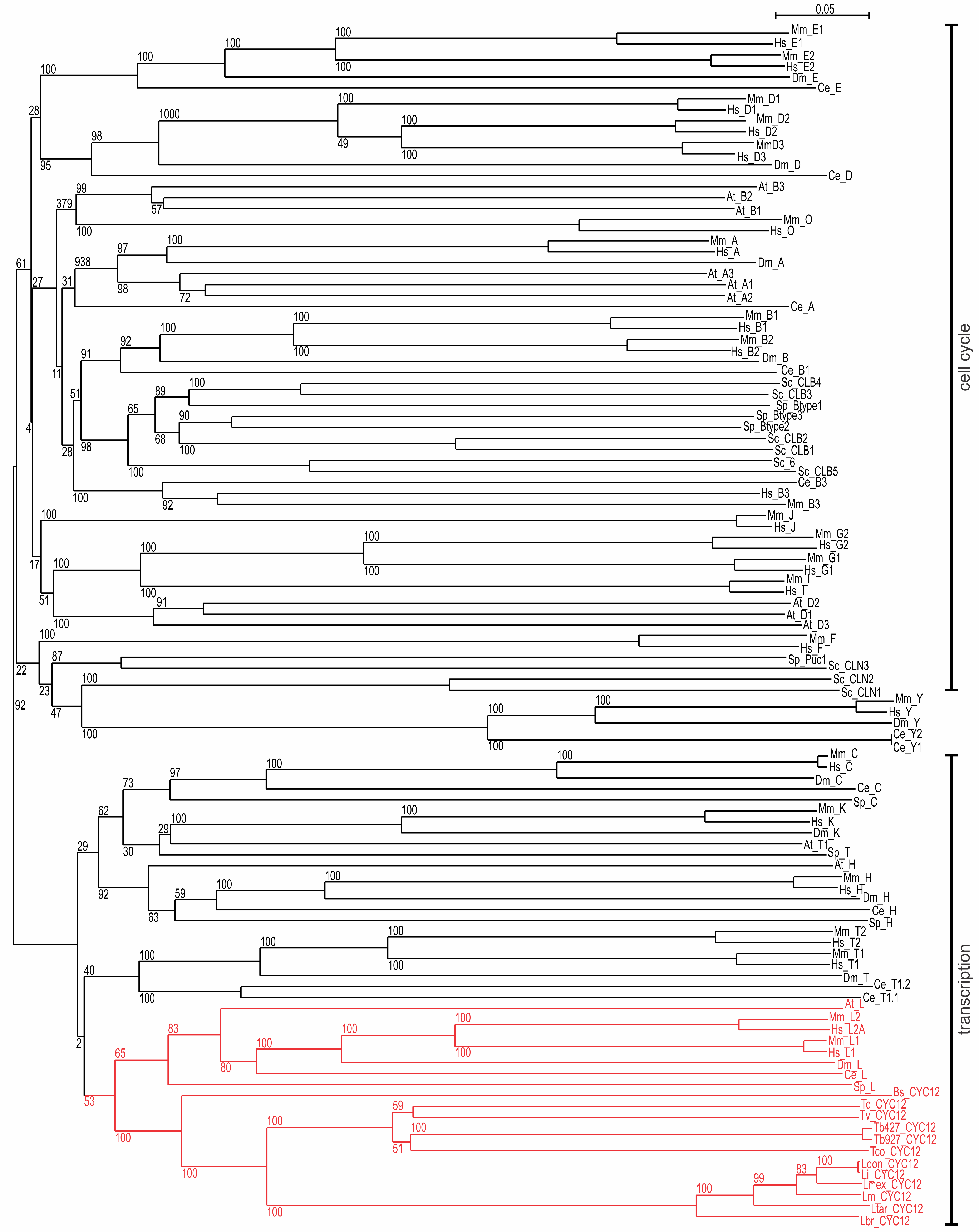

Supplement: S4 Fig — Amino acid sequences of cyclins from H. sapiens (Hs), M. musculus (Mm), Drosophila melanogaster (Dm), C. elegans (Ce), A. thaliana (At), S. cerevisiae (Sc), and S. pombe (Sp) as well as kinetoplastid CYC12 sequences from Trypanosoma brucei brucei strains 427 (Tb427) and 927 (Tb927), Trypanosoma congolense (Tco), Trypanosoma vivax (Tv) T. cruzi (Tc), L. major (Lm), Leishmania infantum (Li), Leishmania donovani (Ldon), Leishmania mexicana (Lmex), Leishmania tarentolae (Ltar), Leishmania braziliensis (Lbr), and the bodonid B. saltans (Bs) were aligned using the Clustal Omega server at http://www.ebi.ac.uk [7]. The multiple sequence alignment was imported into the ClustalX software package [8] and phylogenetically analyzed using the neighborhood joining method. Bootstrap values were obtained by sampling a thousand replicates and are indicated as percentages. The node for the cyclin L/CYC12 cluster is drawn in red. Cyclin clusters of cell cycle-regulating and transcriptional CDKs, according to Ma et al. [9], are indicated. The cyclin sequences were obtained from the following accession numbers: Hs_A (CAA35986.1), Hs_B1 (CAO99273.1), Hs_B2 (AAI05087.1), Hs_B3 (CAC94915.1), Hs_C (AAH41123.1), Hs_D1 (AAH23620.1), Hs_D2 (CAA48493.1), Hs_D3 (AAA52137.1), Hs_E1 (AAH35498.1), Hs_E2 (AAC78145.1), Hs_F (AAB60342.1), Hs_G1 (AAC78145.1), Hs_G2 (AAC41978.1), Hs_H (AAA57006.1), Hs_I (AAF43786.1), Hs_J (AAH43175.1), Hs_K (AAH43175.1), Hs_L1 (AAH43175.1), Hs_L2A (Q96S94.1), Hs_O (NP_066970.3), Hs_T1 (AAC39664.1), Hs_T2 (AAW56073.1), Hs_Y (AAH94815.1), Mm_A (CAA81331.1), Mm_B1 (AAH85238.1), Mm_B2 (AAH08247.1), Mm_B3 (AAI38356.1), Mm_C (AAH03344.2), Mm_D1 (AAO13813.1), Mm_D2 (AAH49086.1), Mm_D3 (AAC53363.1), Mm_E1 (AAI38663.1), Mm_E1 (AAC80527.1) Mm_F (AAA63152.1), Mm_G1 (AAC42082.1), Mm_G2 (AAC32372.1), Mm_H (AAH38861.1), Mm_I (AAF43391.1), Mm_J (AAI20923.1), Mm_K (AAH27297.1), Mm_L1 (AAH94383.1), Mm_L2 (AAI32296.1), Mm_O (AAI47760.1), Mm_T1 (AAD13656.1), Mm_T2 (AAH54122.1), Mm_Y (NP_08 [file ppat.1005498.s004.tif]

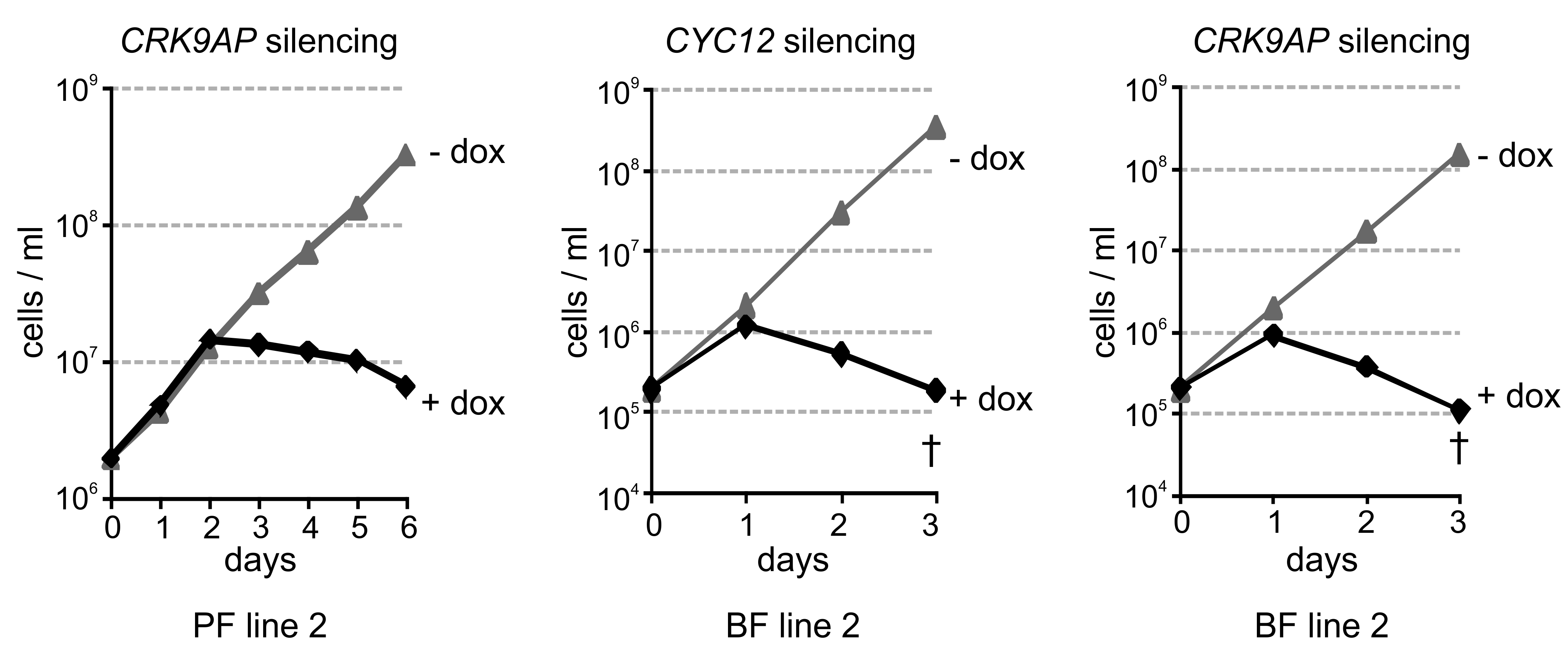

Supplement: S5 Fig — Growth curves of additional PF and BF cell lines in which either CYC12 or CRK9AP was conditionally silenced by doxycycline (dox)-induced dsRNA synthesis. The cross indicates that no viable cells were detectable after day 3 of induction. Corresponding cell lines were analyzed in detail as shown in Fig 4 and S6 Fig. (TIF) [file ppat.1005498.s005.tif]

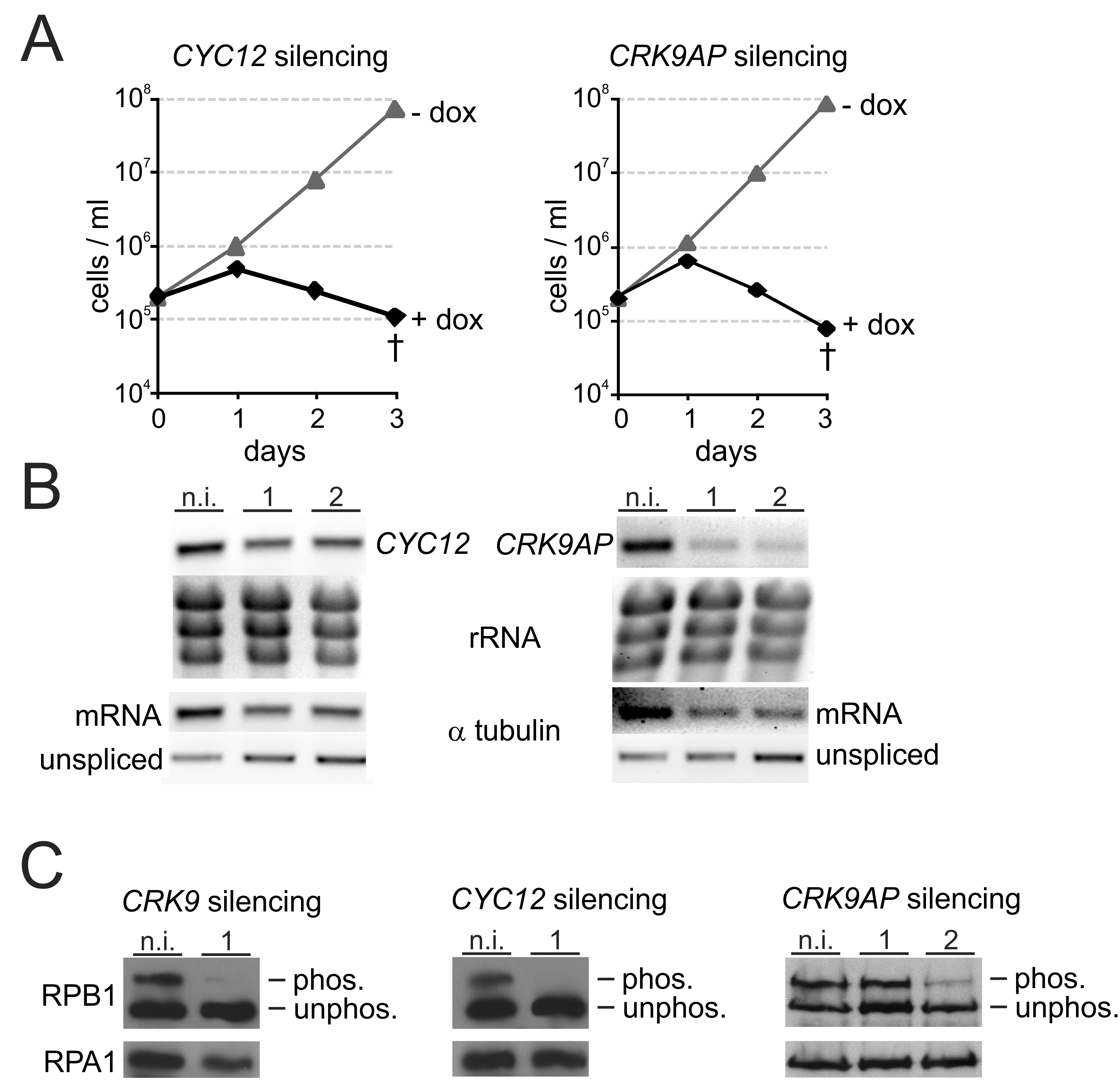

Supplement: S6 Fig — (A) Growth curves of uninduced (- dox) BF cultures or cultures in which CYC12 or CRK9AP was conditionally silenced by the addition of doxycyline to the medium (+ dox). The cross indicates that subsequent to day 3 of induction no intact cells were detectable by microscopic inspection. (B) RNA analyses. Total RNA was prepared from [un-]induced cells and the relative amounts of mature CYC12/CRK9AP and α tubulin mRNA was determined by reverse transcription using an oligo-dT primer and semi-quantitative PCR, performed in the linear range of the amplification reaction. Unspliced, α tubulin pre-mRNA was analyzed by reverse transcription of the same total RNA preparations using random hexamers and PCR with an oligonucleotide that hybridized upstream of the α tubulin SL addition site. rRNA served as a loading control and was detected after RNA separation on agarose gel by ethidium bromide staining. (C) CRK9 silencing in BFs causing a SL trans splicing defect was published previously [10]. Here, immunoblotting of whole cell lysates from these cells shows that RPB1 phosphorylation was lost after one day of induction (unphos.). Corresponding results were obtained when CYC12 or CRK9AP was silenced, although RPB1 dephosphorylation in CRK9AP-silenced cells was consistently observable only after two days of induction. (TIF) [file ppat.1005498.s006.tif]

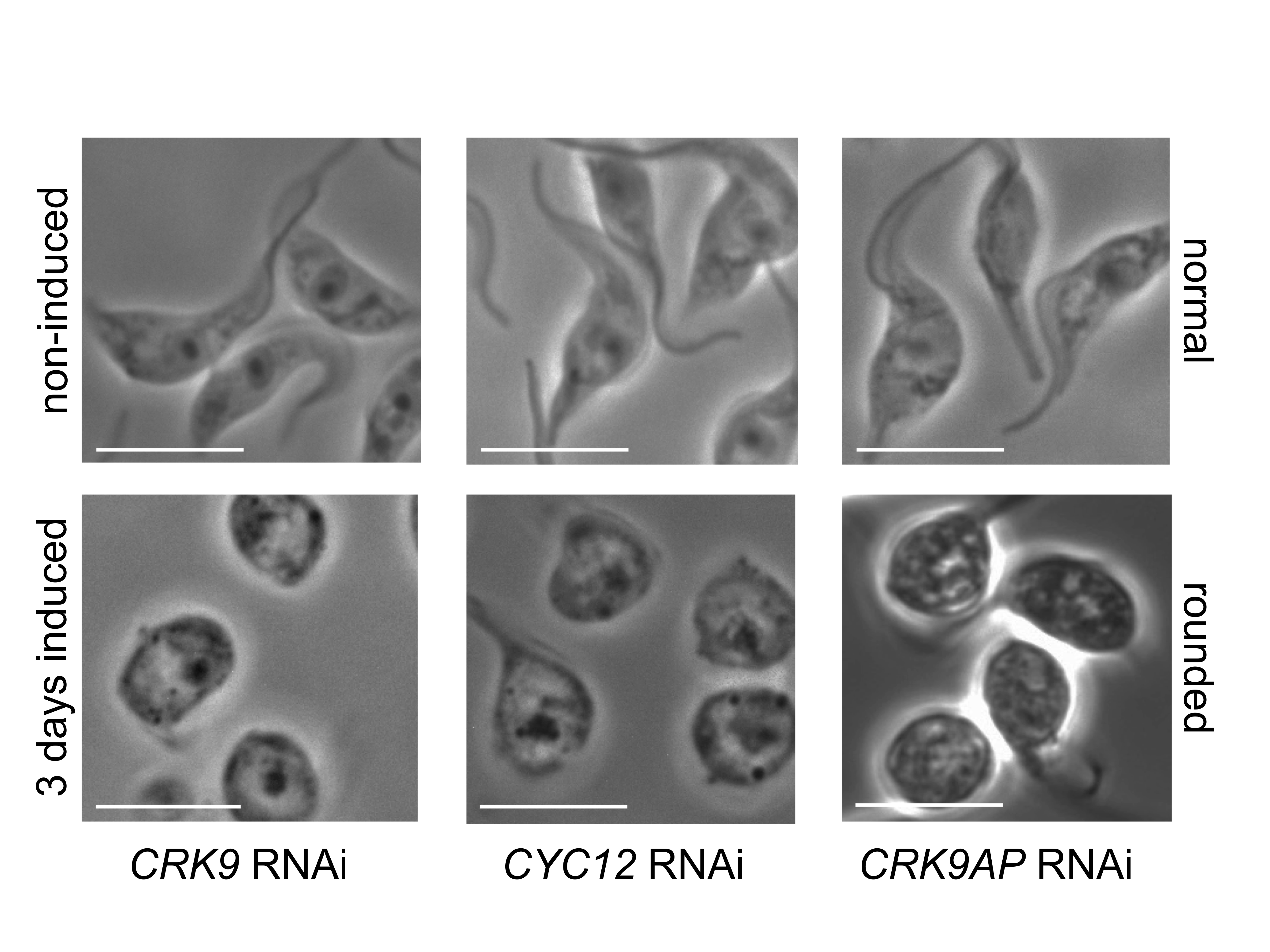

Supplement: S7 Fig — Microscopic images of un-induced procyclic cells that look normal (top row) and of rounded cells from the same cell lines (bottom row) when CRK9, CYC12 or CRK9AP was depleted for three days. White scale bars correspond to 10 μm. (TIF) [file ppat.1005498.s007.tif]

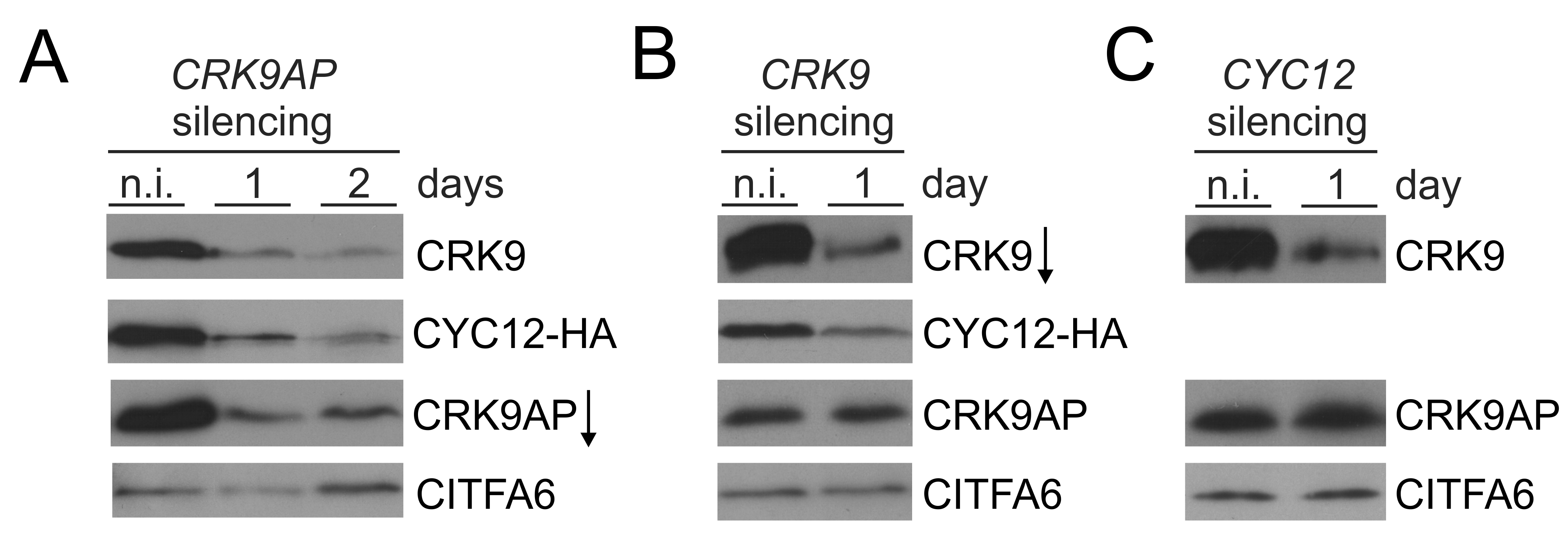

Supplement: S8 Fig — Immunoblotting of whole cell lysates prepared from BFs in which CRK9AP (A), CRK9 (B) or CYC12 (C) were silenced, detecting CRK9, CRK9AP and, as a loading control, the transcription factor subunit CITFA6 with specific polyclonal immune sera and CYC12-HA with a monoclonal anti-HA antibody. Note that silencing specificity and efficiency for the CYC12 RNAi cell line, which harbored immunologically undetectable, endogenous CYC12, was demonstrated on the RNA level in S6 Fig. The protein profile of silenced genes is indicated by an arrow. n.i., non-induced. The experiment shows that CYC12 and CRK9 are lost when other CRK9 complex subunits are depleted whereas CRK9AP abundance remained unaffected and only diminished when the CRK9AP gene was silenced. (TIF) [file ppat.1005498.s008.tif]

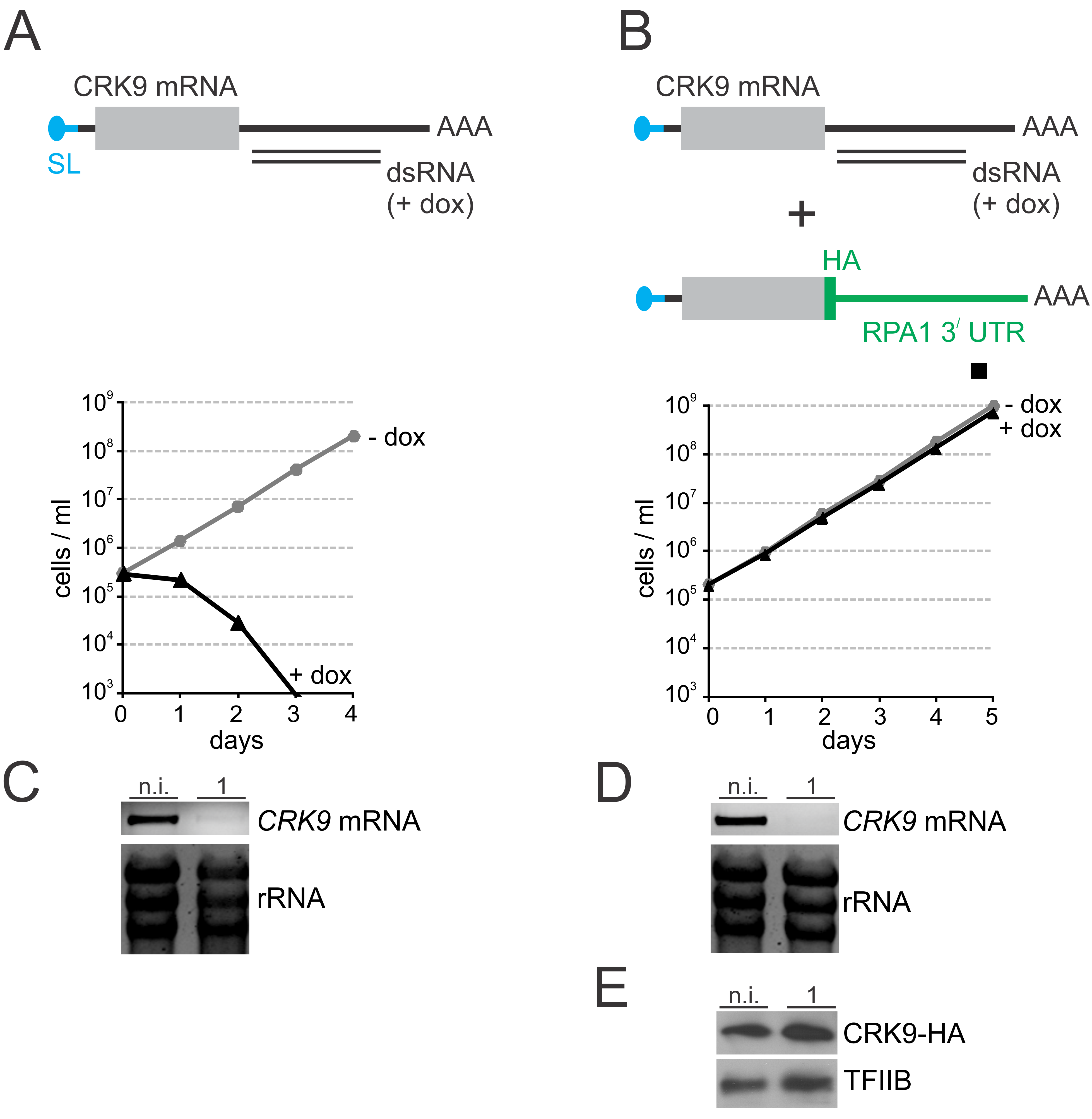

Supplement: S9 Fig — (A and B, top) Schematics reproduced from Fig 7, depicting targeting and rescue of CRK9 mRNA in the two smBF cell lines that were used in mouse infections studies. Culture growth curves on the bottom are from non-induced cells (- dox) and doxycycline-induced (+ dox) trypanosomes of the corresponding cell lines. (C and D) Semi-quantitative RT-PCR analysis of CRK9 mRNA in total RNA preparations from non-induced (n.i.) and one day-induced cells, demonstrating efficient CRK9 silencing. rRNA, visualized by ethidium bromide staining, served as a control for RNA input. (E) Anti-HA immunoblot showed that CRK9-HA protein, expressed from the RNAi-resistant transgene, was unaffected by addition of doxycycline. TFIIB was probed on the same blot as a loading control. (TIF) [file ppat.1005498.s009.tif]
