# Supplementary material for: Cyclin-Dependent Kinase CRK9, Required for Spliced Leader trans Splicing of Pre-mRNA in Trypanosomes, Functions in a Complex with a New L-Type Cyclin and a Kinetoplastid-Specific Protein
Source: PLoS Pathog. 2016 Mar 8;12(3):e1005498. doi: 10.1371/journal.ppat.1005498 (PMC4783070; doi:10.1371/journal.ppat.1005498)
Supplement: S3 Fig — Cyclin L and CYC12 sequences were aligned using the Clustal Omega server at default parameters. Shown are the sequences of the CCL1 domain (COG5333) as defined in human cyclin L1 according to the Conserved Domain Database [6]. Dashes indicate that a corresponding residue is missing. Numbers in parentheses specify number of residues without significant sequence similarity. Positions with more than 50% identity or similarity are highlighted in black or gray, respectively. Identical positions in model organisms without similarity in kinetoplastids were highlighted in blue and insertions or unique identical positions in kinetoplastids were highlighted in red. Stars and colons denote positions that are identical or similar in all sequences analyzed. Yellow highlighting indicates cyclin folds 1 and 2 within the CCL1 domain and question marks indicate positions of the human cyclin L1 sequence that were not recognized as part of the CCL1 domain. Cyclin L sequences are from Homo sapiens (Hs; L1, accession number NP_064703; L2A, NP_112199), Mus musculus (Mm, NP_064321), Danio rerio (Dr, NP_956034), Caenorhabditis elegans (Ce, NP_506007), Arabidopsis thaliana (At, NP_565622) and Schizosaccharomyces pombe (Sp, NP_593045). Kinetoplastid CYC12 sequences were from T. brucei (Tb, accession number Tb927.10.9160), Trypanosoma cruzi (Tc, TcCLB.503525.20), Leishmania major (Lm, LmjF.36.5640), and the bodonid Bodo saltans (Bs, BS70770.1). (DOCX) [file ppat.1005498.s003.docx]

**.xx. |xxxxxxxxxxxxxxxxx Cyclin_N domain xxx xxxxxxxxxxxxxxxxxxxxxxxxx**

***Hs*L1 74 LDLPSETDLRILGCELIQAAGILLRLPQVAMATGQVLFHRFFYSKS[00]FVKHSFEIVAMACINLASKIEEAPR**

***Hs*L2A 69 LDTDTETDLRVVGCELIQAAGILLRLPQVAMATGQVLFQRFFYTKS[00]FVKHSMEHVSMACVHLASKIEEAPR**

***Mm*L 80 LDLPSETDLRILGCELIQAAGILLRLPQVAMATGQVLFHRFFYSKS[00]FVKHSFEIVAMACINLASKIEEAPR**

***Dr*L 54 LDHETETDLRILGCERIQSAGILLRLPQVAMATGQVIFQRFFFSKS[00]FVKHNFEIVAMACVNLASKIEESPR**

***Ce*L 97 LSKETESELRYLGCELIQQGAILLKLPQTAAATGQILFQRYYYQKS[00]FVRYHFEHAVQACLLLASKIEEEPR**

***At*L 26 IDETTEISLRIYGCDLIQEGGILLKLPQAVMATGQVLFQRFYCKKS[00]LAKFDVKIVAASCVWLASKLEENPK**

***Sp*L 19 DSFEYAEELCTLGSEWIQEAGVLLNLTQNCVIVCLILFRRYCTLYP[00]PRVPDLDAIVMACVSIGSKTTETPA**

***Tb*CYC12 9 WLCDARRAFCAYGVDLIRTGSILVRTTPSVTYRASVLFQRFQAAAE[74]EDHDDIVYLAAACILVAAKVEDPST**

***Tc*CYC12 7 WIDDARRGYSSYGVDLIRTGCLLLRTTPSVTYRAAVLFQRFQAVAE[64]REHDDITYLTAACVLIATKMEDHSM**

***Lm*CYC12 2 TDAAARDAYTAYGVDLIRTTGLLLRTTPSTIYRASILFQRFEASVE[93]SDHEDIMYLVAACLLIATKMEDPST**

***Bs*CYC12 22 SAARADQIFNTYTADLLRTLCCLMGVGPTVVFRAQALFQRLTTIVK[50]FTDDSMLYFVAAVVLITCKLECPQA**

***Bs* 22 11111111111111: ::1111*:111111111111:*:*111111111111111111111:1:1:11*111111**

***..* xxxxxxxxx| |?----------?-??????????| |xxxxxxxxxx**

***Hs*L1 145 RIRDLINVFHHLRQLRGKRTPS-----------P-LILDQNYINTKNQVIKAERRVLKELGFCVHVKHPHKIIVM**

***Hs*L2A 140 RIRDVINVFHRLRQLRDKKKPV-----------P-LLLDQDYVNLKNQIIKAERRVLKELGFCVHVKHPHKIIVM**

***Mm*L 151 RIRDVINVFHHLRQLRGKRTPS-----------P-LILDQNYINTKNQVIKAERRVLKELGFCVHVKHPHKIIVM**

***Dr*L 125 RVRDVINVFHHLKQGKGKK-ST-----------P-LILDQNYINTKNQVIKAERRILKELGFCVHVKHPHKIIVM**

***Ce*L 168 RPREVYNVFHRLERLHRLQQSGHDINKETTRGMKPPAVDMNYINTKQHMINSERRILATLGFVVHVKHPHRLIVA**

***At*L 97 KARQVIIVFHRMECRRENLPLE-----------HLDMYAKKFSELKVELSRTERHILKEMGFVCHVEHPHKFISN**

***Sp*L 90 SVQDICNVVVYLKERFKDTNF------EARGFIAHDLYSEEMYSSRNRLSNMELEVLRALNFDTHIVIPHKLAIH**

***Tb*CYC12 154 RIRSIVSVFMRLNQRRRNEPVI--------E--LLQPPPERYENFKTRVREAEEIVLQTLGFQTFVECPFKYAII**

***Tc*CYC12 142 RVRLIVGVFMRLNQRRRGEPVI--------E--QLQPPPERYDDFKSCILEAEEVVLQALGFQTFVESPFKYAIL**

***Lm*CYC12 166 RIRAVVNVCMRLSLRRSGIPVT--------E--QSKPSLPRYEDFKACVIEAEEVVLHQLGFQTFVESPYKYVLL**

***Bs*CYC12 143 KIRLVVHAVHRLNYRRKQRTPVSGSLAAAAT--LPPISPEVYDALKRQVVQAEWIVLVQLGFQVTVECPHKFVFP**

**111111111 111:1:111111:111111111111111111111111111111111:11:111*11:*11:1*111:11*1:111111**

**.. xxxxxxx?-?x----xxx Cyclin_C domain xxxxxxxxxxxxxxxxxxxxxxxxxxxx|**

***Hs*L1 208 YLQVLECE-RN[00]QTLVQTAWNYMNDSLRTNVFVRFQPETIACACIYLAARALQIPL--PTR-PHWFLLFGTT**

***Hs*L2A 203 YLQVLECE-RN[00]QHLVQTSWNYMNDSLRTDVFVRFQPESIACACIYLAARTLEIPL--PNR-PHWFLLFGAT**

***Mm*L 214 YLQVLECE-RN[00]QTLVQTAWNYMNDSLRTNVFVRFQPETIACACIYLAARALQIPL--PTR-PHWFLLFGTT**

***Dr*L 187 YLQVLECE-KN[00]QMLVQTAWNYMNDALRTSAFVRFEPETIACACIYLAARVLQIPL--PSK-PHWFLLFGAT**

***Ce*L 273 YGHTLGITQSR[00]PDILQRSWNYMNDGLRTDIFMRYKPETIACACIFLAARTVENPIALPSTPFHWFEAFDTS**

***At*L 161 YLATLETP---[00]PELRQEAWNLANDSLRTTLCVRFRSEVVACGVVYAAARRFQVPL--PEN-PPWWKAFDAD**

***Sp*L 159 YLQTLQLI-DN[00]KKLLQITWNFLNDASRTRLCVLYPPFSLACGCIAMAARVIGMKL-----PKDWYRVFDTT**

***Tb*CYC12 219 FLGILVGNDPV[45]KKWLADAVCWLNDIPRWRELYAEEAYVLAVCALYWTRPPD-----VSGLPEEWTAAFGVE**

***Tc*CYC12 207 FLGMLIEEDKV[25]KKWLADAVSWLNDIPRCVELYAEEAHVLAVCSLFATRPSN-----ITALPDNWSLAFGLE**

***Lm*CYC12 231 YLNILSEPASD[38]TQWMIRAVQVVNDLPRCRRLLAVPADALAIYAIQQSCPPD-----L-TLPDKWSTAFGVS**

***Bs*CYC12 216 FLAFMIDRSAS[07]VSWSMEACKWLNDCSRFADVNSFDAPLLACVALETTQPME----ATAGLPMLWYVAFGIS**

**1111111111111:111:1111111111111111:1111**11*11111111111:*111:11:1111111111111111*111*111**
