# Supplementary material for: Cyclin-Dependent Kinase CRK9, Required for Spliced Leader trans Splicing of Pre-mRNA in Trypanosomes, Functions in a Complex with a New L-Type Cyclin and a Kinetoplastid-Specific Protein
Source: PLoS Pathog. 2016 Mar 8;12(3):e1005498. doi: 10.1371/journal.ppat.1005498 (PMC4783070; doi:10.1371/journal.ppat.1005498)
Supplement: S2 Table — SI_Caption> (DOCX) [file ppat.1005498.s011.docx]

Table S2. List of oligonucleotides used in RNA analysis

| **Purpose** | **Description** | **Sequence** |
| --- | --- | --- |
| RT-[q]PCR | *CYC12* mRNA | **5/-GTAACACCGCCATCGTTGGCGG-3/**  **5/-CTCCACCCGGCAATACCTCCTC-3/** |
| RT-[q]PCR | *CRK9AP* mRNA | **5/-GATAAGCTTACGCGTTAGGCTGACGAGTTTGATG-3/**  **5/-GATCTAGACTCGAGGCGTGCACACAAACCTCTC-3/** |
| RT-[q]PCR | *CRK9* mRNA | **5/-GATAAGCTTACGCGTGTTGTTAAGAAAGAAAATTGA  GG-3/**  **5/-GATCTAGACTCGAGCGCCGCCGCTGCCAACCCCACTC-3/** |
| RT-PCR | α tubulin pre-mRNA | **5/-GTAAGTGGTGGTGGCGTAAG-3/**  **5/-CAATGTGGATGCAGATAGCC-3/** |
| RT-PCR | α tubulin mRNA | **5/-ACAGTTTCTGATCTATATTGATCTT-3/**  **5/-GAGAGTTGCTCGTGGTAGGC-3/** |
| RT-PCR | *RPB7* pre-mRNA | **5/-CCACTCGAAGGAGTAGTTTTC-3/**  **5/-TTATGTGCACTTGCTGGTG-3/** |
| RT-PCR | *RPB7* mRNA | **5/-CATGGGCCCGAGAGGAATATAAAAGTGGAGCCTC-3/**  **5/-ATTCTGATTTGTGCGGGC-3/** |
| RT-qPCR | 18S rRNA | **5/-TCATCAAACTGTGCCGATTAC-3/**  **5/-CTATTGAAGCAATATCGG-3/** |
| Primer extension | SL_PE (SL RNA)  U2f (U2 snRNA) | **5/-CGACCCCACCTTCCAGATTC-3/**  **5/-ACAGGCAACAGTTTTGATCC-3/** |

For each PCR primer pair, the forward primer is listed on top and the reverse primer on the bottom. The gene-specific sequence is underlined when oligonucleotides carry additional nucleotides.
