# Supplementary material for: Cyclin-Dependent Kinase CRK9, Required for Spliced Leader trans Splicing of Pre-mRNA in Trypanosomes, Functions in a Complex with a New L-Type Cyclin and a Kinetoplastid-Specific Protein
Source: PLoS Pathog. 2016 Mar 8;12(3):e1005498. doi: 10.1371/journal.ppat.1005498 (PMC4783070; doi:10.1371/journal.ppat.1005498)
Supplement: S1 References — (DOCX) [file ppat.1005498.s012.docx]

**S1 References**

1. Sievers F, Wilm A, Dineen D, Gibson TJ, Karplus K, Li W, et al. (2011) Fast, scalable generation of high-quality protein multiple sequence alignments using Clustal Omega. Mol Syst Biol 7: 539. PMID: 21988835 doi: 10.1038/msb.2011.75.

2. Marchler-Bauer A, Lu S, Anderson JB, Chitsaz F, Derbyshire MK, Weese-Scott C, et al. (2011) CDD: a Conserved Domain Database for the functional annotation of proteins. Nucleic Acids Res 39: D225-D229. PMID: 21109532 doi: 10.1093/nar/gkq1189.

3. Aslett M, Aurrecoechea C, Berriman M, Brestelli J, Brunk BP, Carrington M, et al. (2010) TriTrypDB: a functional genomic resource for the Trypanosomatidae. Nucleic Acids Res 38: D457-D462. PMID: 19843604 doi: 10.1093/nar/gkp851.

4. Siegel TN, Hekstra DR, Wang X, Dewell S, Cross GA (2010) Genome-wide analysis of mRNA abundance in two life-cycle stages of *Trypanosoma brucei* and identification of splicing and polyadenylation sites. Nucleic Acids Res 38: 4946-4957. PMID: 20385579 doi: 10.1093/nar/gkq237.

5. Schimanski B, Brandenburg J, Nguyen TN, Caimano MJ, Günzl A (2006) A TFIIB-like protein is indispensable for spliced leader RNA gene transcription in *Trypanosoma brucei*. Nucleic Acids Res 34: 1676-1684. PMID: 16554554

6. Logan-Klumpler FJ, De SN, Boehme U, Rogers MB, Velarde G, McQuillan JA, et al. (2012) GeneDB--an annotation database for pathogens. Nucleic Acids Res 40: D98-108. PMID: 22116062 doi: 10.1093/nar/gkr1032.

7. McWilliam H, Li W, Uludag M, Squizzato S, Park YM, Buso N, et al. (2013) Analysis Tool Web Services from the EMBL-EBI. Nucleic Acids Res 41: W597-600. PMID: 23671338 doi: 10.1093/nar/gkt376.

8. Larkin MA, Blackshields G, Brown NP, Chenna R, McGettigan PA, McWilliam H, et al. (2007) Clustal W and Clustal X version 2.0. Bioinformatics 23: 2947-2948. PMID: 17846036

9. Ma Z, Wu Y, Jin J, Yan J, Kuang S, Zhou M, et al. (2012) Phylogenetic analysis reveals the evolution and diversification of cyclins in eukaryotes. Mol Phylogenet Evol 66: 1002-1010. PMID: 23261709 doi: 10.1016/j.ympev.2012.12.007.

10. Badjatia N, Ambrósio DL, Lee JH, Günzl A (2013) Trypanosome cdc2-related kinase 9 controls spliced leader RNA cap4 methylation and phosphorylation of RNA polymerase II subunit RPB1. Mol Cell Biol 33: 1965-1975. PMID: 23478263 doi: 10.1128/MCB.00156-13.
